# Biochemical Function, Molecular Structure and Evolution of an Atypical Thioredoxin Reductase from *Desulfovibrio vulgaris*

**DOI:** 10.3389/fmicb.2017.01855

**Published:** 2017-09-29

**Authors:** Odile Valette, Tam T. T. Tran, Christine Cavazza, Elodie Caudeville, Gaël Brasseur, Alain Dolla, Emmanuel Talla, Laetitia Pieulle

**Affiliations:** ^1^Aix-Marseille Univ, CNRS, LCB, Marseille, France; ^2^Laboratoire de Chimie et Biologie des Métaux, Université Grenoble Alpes, Grenoble, France; ^3^UMR 5249, Laboratoire de Chimie et Biologie des Métaux, Centre National de la Recherche Scientifique, Grenoble, France; ^4^DRF/BIG/CBM, CEA-Grenoble, Grenoble, France

**Keywords:** thioredoxin reductase, oxidative stress, crystal structure, phylogenomics, *Desulfovibrio*, anaerobes

## Abstract

Thioredoxin reductase (TR) regulates the intracellular redox environment by reducing thioredoxin (Trx). In anaerobes, recent findings indicate that the Trx redox network is implicated in the global redox regulation of metabolism but also actively participates in protecting cells against O_2_. In the anaerobe *Desulfovibrio vulgaris* Hildenborough (*Dv*H), there is an intriguing redundancy of the Trx system which includes a classical system using NADPH as electron source, a non-canonical system using NADH and an isolated TR (DvTRi). The functionality of DvTRi was questioned due to its lack of reactivity with DvTrxs. Structural analysis shows that DvTRi is a NAD(P)H-independent TR but its reducer needs still to be identified. Moreover, DvTRi reduced by an artificial electron source is able to reduce in turn DvTrx1 and complexation experiments demonstrate a direct interaction between DvTRi and DvTrx1. The deletion mutant *tri* exhibits a higher sensitivity to disulfide stress and the gene *tri* is upregulated by O_2_ exposure. Having DvTRi in addition to DvTR1 as electron source for reducing DvTrx1 must be an asset to combat oxidative stress. Large-scale phylogenomics analyses show that TRi homologs are confined within the anaerobes. All TRi proteins displayed a conserved TQ/NGK motif instead of the HRRD motif, which is selective for the binding of the 2′-phosphate group of NADPH. The evolutionary history of TRs indicates that *tr1* is the common gene ancestor in prokaryotes, affected by both gene duplications and horizontal gene events, therefore leading to the appearance of TRi through subfunctionalization over the evolutionary time.

## Introduction

The maintenance of a proper disulfide state in the extracellular or intracellular environment is crucial for cell survival. As reductants of intracellular disulfides, the principal protein systems that control the thiol/disulfide redox balance in the cytoplasm are the NADPH-dependent-TR/Trx system and the glutathione reductase/glutathione/glutaredoxin pathway. The TR/Trx system is widespread in all three domains of life, while the glutathione pathway is absent in many microorganisms, including some anaerobic bacteria (Hirt et al., 2002; Hummel et al., 2005; Newton et al., 2009; Pieulle et al., 2011). Trx is a small redox-active protein (∼12 kDa), which has a conserved CxxC catalytic site that undergoes reversible oxidation/reduction of both cysteine residues whereas TR is a member of the flavoprotein family of pyridine nucleotide disulfide oxidoreductases and functions in a homodimeric form. Within each TR monomer, reducing equivalents are transferred to Trx from NADPH *via* a tightly TR-bound flavin adenine dinucleotide (FAD) and a redox-active disulfide (CxxC motif). A peculiar feature of TR from prokaryotes, plants and yeasts (named low *M*_r_ TR of ∼70 kDa) is that the redox-active disulfide is not located in the FAD domain. Consequently, a change of conformation is required for the catalysis. In the FO conformation, the flavin and the disulfide were juxtaposed for flavin oxidation and the redox-active disulfide pair is in a buried state, forbidding direct interaction with Trx (Lennon et al., 2000). Alternatively, in the 67°-rotated FO conformation, named FR conformation, the flavin and the pyridine nucleotides were juxtaposed to allow flavin reduction (Mulrooney and Williams, 1997; Veine et al., 1998; Lennon et al., 2000). The FR conformation is also required for the reduction of the large protein substrate Trx by the TR dithiol.

The universality of the TR/Trx system can be explained by the function of reduced Trx in vital cellular processes as the reduction of nucleotides to deoxynucleotides in bacteria or the modulation of transcription factors in eukaryotes (Holmgren et al., 2005; Toledano et al., 2007). Moreover, the TR/Trx system is a major line of cellular defense against oxygen damage, regenerating oxidative damaged proteins, modulating the activity of redox stressors and acting as hydrogen donors for detoxification enzymes that are important during the oxidative stress response (Zeller and Klug, 2006; Toledano et al., 2007). During the last decade, an increased interest in the TR/Trx system from anaerobic organisms, revealed a significant number of publications, focusing on both the characteristics of Trx and TR as well as their physiological roles (Hammel et al., 1983; Lee et al., 2000; Kashima and Ishikawa, 2003; Sarin and Sharma, 2006; Hernandez et al., 2008; Hosoya-Matsuda et al., 2009; Reott et al., 2009; Yang and Ma, 2010; Pieulle et al., 2011; Garcin et al., 2012; McCarver and Lessner, 2014; Susanti et al., 2014, 2016; McCarver et al., 2017). The properties and roles described for anaerobes confirm and extend the properties established for aerobic forms of life. For example, in the strict anaerobe *Bacteroides fragilis*, a critical role in the redox homeostasis and survival during oxidative stress has been revealed (Reott et al., 2009). In the same way, in sulfate-reducing bacteria (SRB), the pyruvate-ferredoxin oxidoreductase (PFOR), a key enzyme of anaerobic metabolism, is activated by Trx after an oxidative stress (Vita et al., 2008; Pieulle et al., 2011). Moreover, the recent discovery of class III anaerobic ribonucleotide reductases that use the Trx system for the biosynthesis of dNTPs supported the essential function of Trx in anaerobes (Wei et al., 2014). Beside the functional aspect, studies of Trx systems in anaerobes also allowed to uncover some new properties. The TR of the hyperthermophilic bacterium *Thermotoga maritima* showed a preference for NADH over NADPH and could also catalyze the reduction of 5,5′-dithio-bis(2-nitrobenzoic acid) (DTNB) directly which is a characteristic of eukaryotic TR (Yang and Ma, 2010). The saccharolytic fermenter *Clostridium pasteurianum* uses reduced ferredoxin (Fd) instead of NADPH for Trx reduction and transfer of electrons (Hammel et al., 1983). The use of Fd is common to another specific TR that contains iron-sulfur cluster instead of FAD (Jacquot et al., 2009). Similarly, the TR from *Thermoplasma acidophilum* shares overall structural and redox properties with canonical TR but lacks the appropriate binding motif VxxxHRRDxxRA to use NADPH as reductant (Hamill et al., 2008; Hernandez et al., 2008). In this context, studies of the TR/Trx system of SRB, revealed the presence of the canonical bacterial system named DvTR1/DvTrx1 and a second novel system named DvTR3/DvTrx3 (Pieulle et al., 2011). DvTR3 uses preferentially NADH instead of NADPH while DvTrx3 did not exhibit a protein disulfide reductase activity but an unusual disulfide isomerase activity *in vitro* (Garcin et al., 2012). A third TR named DvTRi (i for isolated) was identified in *Desulfovibrio vulgaris* Hildenborough (*Dv*H) (Heidelberg et al., 2004; Pieulle et al., 2011). DvTRi is encoded by a monocistronic operon without a closely associated Trx encoding gene, in contrast to the DvTR1 and DvTR3 genes. Moreover, we previously established that no DTNB reduction occurred when the reductase DvTRi was used with either DvTrx1 or DvTrx3 as redox partners (Pieulle et al., 2011). Since these Trxs are most likely the only cytoplasmic thiol/disulfide oxidoreductases of *Dv*H (Pieulle et al., 2011), the results raised the question about the function of DvTRi.

In this work we combined enzymatic, genetic, structural and phylogenomic approaches in order to address this question. Enzymatic and structural characterization of DvTRi clearly shows that NAD(P)H does not serve as electron donor for the FAD while DvTrx1 was identified as the substrate. A phenotypic analysis of the *Dv*H *tri* deletion mutant revealed that DvTRi contributes with the DvTR1/Trx1 system to the control of the thiol/disulfide homeostasis during oxidative conditions. In addition, we show that members of the TRi subfamily exhibit specific patterns that may explain the functional relationships between these proteins and their reductants. Finally, phylogenomics approach led us to conclude that TRi and other TR types have a distinct and complex evolutionary history, mainly driven by duplications and horizontal gene transfer (HGT).

## Materials and Methods

### Bacterial Strains and Growth Conditions

*Dv*H was grown anaerobically at 33°C in lactate/sulfate modified LS4D medium (Mukhopadhyay et al., 2006). The pH of the modified LS4D medium was adjusted to 7.4 with 10 M NaOH after addition of all medium components: 8 mM magnesium chloride, 20 mM ammonium chloride, 0.6 mM calcium chloride, 2.2 mM potassium phosphate (dibasic), 30 mM Pipes buffer, 1 ml Thauer’s vitamin solution (Badziong and Thauer, 1978), and 12.5 ml trace element solution per liter. Trace element solution contained 2.5 mM manganese chloride, 1.26 mM cobaltous chloride, 1.47 mM zinc chloride, 210 μM sodium molybdate, 320 μM boric acid, 5 mM iron chloride and 67 mM nitrilotriacetic acid. Sodium lactate (47 mM) was added as the electron donor and sodium sulfate (30 mM) as the terminal electron acceptor. For solid medium, 15 g per liter of agar was added. When noted, yeast extract (1 g/liter) was added and the medium was designated LS4DYE medium. Antibiotics were added to the medium when required as follows: kanamycin (Kan) at 50 μg/mL, thiamphenicol (Tm) at 20 μg/mL and gentamycin (Gm) at 200 μg/mL. It is noteworthy that *Dv*H is able to resist to kanamycin at 50 μg/mL. However, for growth analyses and diamide sensitivity assays, Kan and Tm were omitted. The monitoring of cell growth was followed through the optical density at 600 nm with a spectrophotometer (CO8000 Cell Density Meter, Labgene Scientific Instruments, France). Cultures were inoculated at 10% (v/v) in 10 mL (Hungate tubes) with cells in the stationary growth phase. For each growth assay, four biological replicates were carried out.

### Production and Purification of the DvTR and DvTrx

Wild-type DvTR and DvTrx were heterologously produced in *E. coli* (*Ec*) and purified as reported in Pieulle et al. (2011). DvTRi and DvTrx1 variants were produced as follows: primers, plasmids and *Ec* strains used for the production of the variants are reported in Supplementary Table S1. The mutagenic PCR products were obtained by overlap extension PCR and directly introduced into chemically competent *Ec* DH5α cells. Transformants were selected as ampicillin (100 μg/mL)-resistant colonies. The mutagenic plasmids obtained were then transformed in *Ec* TG1 for the production. The production and the purification of the four variants were performed as reported previously (Pieulle et al., 2011). To improve the purification of Trx1C33S, an additional step, before the heating of the proteins fraction, was necessary. This fraction was incubated during 1 h with 100 mM DTT in order to eliminate the *Ec* proteins linked through a mixed disulfide bridge involving C31 of DvTrx1. DTT was eliminated by a dialysis onto a Zeba spin desalting column (Thermo Scientific). The purity of DvTRiC131S, DvTRiC134S, DvTrx1C30S and DvTrx1C33S were checked on a SDS-PAGE (Supplementary Figure S1A) and the presence of FAD in the TRi variants was controlled using a Beckman DU40 spectrophotometer (Supplementary Figure S1B). The protein concentration was measured using the BCA protein assay kit (Pierce) and TRi concentration was also calculated using the 𝜀_451 nm_ value of 11,300 M^-1^cm^-1^.

### Reduction of DvTRs by NAD(P)H

DvTR proteins were incubated in an anaerobic glass cuvette with 100 μM NAD(P)H at 25°C in 50 mM Tris-HCl, pH 7.5 and 150 mM NaCl. All the experiments were performed under anaerobic conditions in order to prevent the reoxidation of FAD by oxygen. Spectra were recorded from 650 to 240 nm with an Uvikon 930 spectrophotometer to monitor the reduction of the enzyme-bound flavin.

### Benzyl Viologen Oxidoreductase Activity

Benzyl viologen oxidoreductase (BVOR) activity was determined in an anaerobic glass cuvette by monitoring the NAD(P)H-dependent benzyl viologen (BV) reduction at 580 nm (𝜀_580_ = 8.8 mM^-1^cm^-1^) at 30°C. The assay mixture (1 mL) contained 1 mM BV and 1 mM NADPH or NADH in 100 mM KPO_4_ buffer, pH 7.5. One unit of BVOR activity was defined as 2 μmol BV reduced per min. A final concentration of 0.1, 0.5, and 33 μM was used for DvTR3, DvTR1 and DvTRi, respectively.

### Xanthine/Xanthine Oxidase Reduction System and Gel-Based Oxidation State of Thioredoxin Measurement

To test whether reduced DvTRi was able to reduce DvTrx1 and DvTrx3, the xanthine/xanthine oxidase reduction system was used (Massey et al., 1969). All steps were achieved in a Jacomex anaerobic chamber except for the recording of the spectral features. The DvTRi reduction was performed as follows: from a solution of 100 mM phosphate buffer (pH 7.5) with 1 μM benzyl viologen, 250 μM xanthine and 12 μM DvTRi, an aliquot of xanthine oxidase (final concentration 70 nM) was added to initiate the reaction. After 1 h at 30°C, the sample was transferred into an anaerobic sample cuvette to verify the reduction of bound FAD using an Uvikon 930 spectrophotometer. When the complete reduction of DvTRi was achieved, oxidized DvTrx1 was added to a final concentration of 24 μM, and the spectral features were again monitored.

Reduction of DvTrx1 by DvTR1 was evaluated by the decrease in electrophoretic mobility caused by covalent modification of DvTrx1 by AMS, a thiol-reactive probe when the disulfide is reduced. After the reoxidation of DvTRi by the addition of oxidized DvTrx1 in the reaction mixture, 5 μL of sample were removed and immediately added to 5 μL of 30 mM AMS in TE buffer (pH 7.5). The AMS was allowed to react 15 min at 25°C with reduced sulfhydryl groups (Tibodeau et al., 2009), then the sample was mixed with non-reducing sample buffer and was electrophoresed on 17.5% SDS-polyacrylamide gel. The gels were stained with Coomassie blue. For control assays, all steps were achieved as described above.

### Complexation Reaction between TRi and Trx1 Variants

The procedure used was modified from Wang et al. (1996). DvTrx1C30S, DvTrx1C33S, DvTRiC131S and DvTRiC134S were treated with DTT (25-fold excess) for 60 min at 30°C before the reaction to make sure that the remaining active site thiol was in the reduced state. The protein concentration used was ∼100 μM. Then, DTT was removed onto a Zeba spin desalting column equilibrated with 50 mM Na/K phosphate at pH 8.8, previously degazed. Reduced DvTrx1 variants were incubated with 40-fold excess of DTNB during 1 h at 30°C and subsequently dialyzed to remove DTNB and TNB. DvTRi variants were reacted with equimolar mixed disulfide DvTrx1C-TNB in 50 mM Na/K phosphate (pH 8.8), 2 h at 37°C. DvTRi-DvTrx1 complexes were visualized by western blotting. Protein samples were resuspended in reducing (DTT, 10 mM) or non-reducing loading buffer, heated at 95°C for 10 min and separated on SDS-PAGE. DvTrx1 was detected with rabbit-raised antiserum of EcTrxA diluted in 5% milk (1:5000) followed by horseradish peroxidase (HRP) conjugated goat anti-rabbit IgG (1:5000) for detection by chemiluminescence.

### Construction of the ***Δ****tri* Deletion Mutant and Strains for Complementation Study

Construction of the *Dv*H *Δtri* mutant was achieved as reported recently in (Vita et al., 2015). Briefly, the two ∼500 bp regions upstream and downstream of the *tri* gene, respectively, were cloned into the pNOTCmΔ which produced pNOTCmΔ*tri* (Supplementary Table S1) and the mutagenic plasmid was transferred into *Dv*H by electrotransformation. The colonies appeared after 5–6 days were screened by PCR with the primers reported in Supplementary Table S1 to determine whether the appropriate deletion mutant strain was obtained. To complement the deletion mutant strain, a plasmid that stably replicates in *Dv*H was constructed. An amplicon containing the *tri* gene was obtained by PCR amplification with the primers reported in Supplementary Table S1. The amplicon was digested with *Pst*I and *Kpn*I and ligated to similarly cleaved pBMG6 (Perkins et al., 2011). The produced plasmid, named pG6*tri* (∼500 ng), was transformed into Δ*tri* strain *via* electroporation. In parallel, the plasmid pBMG6 was introduced into WT and Δ*tri* strains.

### Diamide Disk Diffusion Assays

All steps were performed in an anaerobic chamber. Disk diffusion assays were performed by spreading 100 μL of a dilution 10^-2^ of washed cells in the early stationary phase growth (OD_600_∼0.9) on plates with modified LS4D medium, allowing the plates to dry, and then adding a sterile 12-mm filter disk (Whatman^®^) to the center of the plate. Twenty microliters of diamide (1 or 1.5 M) was added to the disk, and then the plates were placed in the incubator at 33°C. Following 6 days incubation, the diameters of the zones of growth inhibition were measured, and the results are the averages of two independent experiments done in triplicate. For each strain, a control with 20 μL of anaerobic milliQ water was performed where no growth inhibition zone was observed.

### Transcriptional Analyses

RNAs were prepared from a *Dv*H culture (40 mL) in the exponential growth phase (OD_600_∼0.5) which was bubbled with air for 1 h, at 33°C. Then, the cells were harvested and resuspended in 200 μl of 10 mM Tris-HCl (pH 8.0) buffer. Total RNA extraction, cDNA synthesis and quantitative real-time PCR (qRT-PCR) analyses were performed as described recently (Vita et al., 2015). Briefly, total RNAs, from three independent biological cultures, were isolated using the High Pure RNA kit from RocheLife Science (Roche Diagnostic, France). The RNA quality was assessed by agarose gel electrophoresis, and the absence of DNA contamination was confirmed by PCR. cDNA synthesis was obtained from 10 μg of RNA quantified spectrophotometrically at 260 nm (NanoDrop 1000; Thermo Fisher Scientific, United States). All of the primer pairs used for qRT-PCR are reported in Supplementary Table S1.

### Crystallization, Data Collection and Structure Determination of DvTRi

Protein crystallization conditions were obtained either manually or by using crystallization screens (Hampton Research Grid Screens^TM^ and Qiagen protein crystallization suites). The crystallization conditions were then manually optimized. Diffracting crystals were obtained in two different conditions: (i) 2 μL of a 10 mg/ml protein solution were mixed with 2 μL of 12% PEG 3350, 100 mM BisTris pH 6.0 reservoir solution (crystals 1); (ii) 2 μL of a 10 mg/ml protein solution were mixed with 2 μL of 25% PEG 4000, 50 mM ammonium acetate, 100 mM sodium citrate pH 5.6 reservoir solution (crystals 2). Yellow crystals appeared in a few days and were cryo-protected using a solution obtained by adding 25% (v/v) glycerol to the mother liquor and flash-cooled in liquid nitrogen. Data were collected at beamlines ID29 and BM-30A of the European Synchrotron Radiation Facility (ESRF) in Grenoble, France. Data reduction was carried out using XDS (Kabsch, 2010) The structure was solved from crystals 1 by molecular replacement, using Balbes which created a partial model lacking the NADPH-binding domain from the X-ray structure of the TR from *Sulfolobus solfataricus* (PDB ID: 3F8D). The NADPH-binding domain was then added in a second step using PHASER (McCoy et al., 2007). Crystallographic refinements were conducted using Phenix (Adams et al., 2010) and the three-dimensional models were examined and modified using the graphics program Coot (Emsley et al., 2010). Crystallographic statistics are summarized in Supplementary Table S2. Superimposition of models and rmsd were calculated using the Secondary-Structure Matching (SSM) tool (Krissinel and Henrick, 2004). **Figure 5** was prepared with PyMol (the PyMOL Molecular Graphics System, Version 1.7, Schrödinger, LLC.).

### Identification of DvTRi, DvTR1 and DvTR3 Specific Homologs As Well As Specific Motifs Associated with These Thioredoxin Reductases

The complete genomes (including their taxonomy lineages) of 2772 prokaryotic (2607 bacterial and 165 archaeal) organisms available in March 2015 were downloaded from NCBI ftp site^1^ and constituted the primary data source. This data was cross-compared with NCBI representative prokaryotic genomes^2^ leading to 1084 representative complete genomes. The list of experimentally known TR proteins used as seed proteins are presented in Supplementary Material (SM). Through the use of HMMER package with Pfam domain database and protein similarity networks (PSN) associated to reference seed proteins, we first established PSN group of homologs for DvTRi, DvTR1 and DvTR3 proteins within representative genomes. Details of the procedures are described in SM.

In order to define specific motifs related to active site/NAD(P)H binding region, specific proteins associated to each PSN group of homologs (here TRi, TR1 or TR3), were first determined through cross-comparison of the group members. Then, for specific PSN group and each protein, PNDO sequence regions were extracted, followed by protein multiple alignment using ClustalW, version 2.1 (Larkin et al., 2007). Firstly, the active site/NAD(P)H binding region was split in two sub-regions (named BoxA and BoxB) in accordance with TR functional elements. Indeed, BoxA contains the pyrophosphate binding site (PP) of NAD(P)H, the CxxC active site and the Trx binding sites (GR/KG and F), and BoxB essentially harbors the phosphate binding site (2′P) of the NADPH. Sizes of BoxA and BoxB sub-regions were arbitrarily set to 31 and 20 amino acids, respectively. Subsequently, sequence logos relative to BoxA and BoxB sub-regions were performed using the Weblogo program (Crooks et al., 2004). In order to enrich the motif and to get more TRi, TR1, and TR3 homologs, we set up an iterative procedure (2 runs) as follows (for each TR group) (Supplementary Figure S2): (i) an HMM profile was built from the BoxB region and used as query to search against the PNDO proteins within all complete prokaryotic genomes with a manually defined trusted cut-off threshold, the later was considered to be the score of the lowest-scoring known true positive (in other words, the minimal bit score observed for PNDO proteins used to build the HMM profile) and (ii) with the obtained TR homologs, the same procedure was then repeated until the number of TR homologs remains constant. In the case of TR1 group of homologs, they were separated in TR1 and FdR specific subgroups of homologs based on the presence and absence of the two conserved cysteine residues within the active site regions, respectively. Similarly, TR3 group of homologs was divided in two specific subgroups: TR3 and dcTR1 (degenerated *Clostridium* TR, dcTR1) based on the presence of the related non-canonical Trx3 within the genome. For that purpose, we performed a blast comparison using DvTrx3 protein (as seed) against clostridial genomes with an *e*-value threshold less than e-10.

### Phylogenetic Analyses

Two different datasets (16S rRNA sequences; protein homologs from TRi, TR1 and dcTR1/TR3 groups) including sequences from Deltaproteobacteria and Clostridia taxonomic classes as well as species from Actinobacteria phylum as outgroup, were used. For the reference tree, 16S rRNA sequences were predicted by RNAmmer 1.2 (Lagesen et al., 2007). The 16S rRNA sequences and TR proteins were aligned using ClustalW, version 2.1 (Larkin et al., 2007), and MUSCLE, version 3.8 (Edgar, 2004), respectively; followed by selection of unambiguous parts of the alignments with Gblocks, version 0.91b (Talavera and Castresana, 2007). In order to get both BoxA and BoxB regions within Gblocks selection sites, Gblocks parameters were modified as follows: a minimal number of sequences within the flank positions higher than 50% of the number of sequences, a minimal length of accepted blocks of 2 positions, and positions with gaps were allowed. Prior to the phylogenetic reconstruction, best-fit models of sequence evolution were selected with jModelTest, version 2.1.10 (Guindon and Gascuel, 2003; Darriba et al., 2012) for the 16S rRNA dataset and ProtTest, version 3 (Darriba et al., 2011) for the TR alignments, respectively. Phylogenetic reconstructions were performed using bayesian inference methods implemented in the MrBayes, version 3.2.6 (Ronquist and Huelsenbeck, 2003; Ronquist et al., 2012) with the GTR substitution model with an estimated proportion of gamma distribution and invariant sites (GTR + I + G) in the case of 16S rRNA or with LG + I + G model for TR proteins. The phylogenetic trees were visualized by FigTree, version 1.4^3^ and were rooted using the outgroup approach.

## Results

### DvTRi Is Not Reduced by NAD(P)H

**FIGURE 1:**
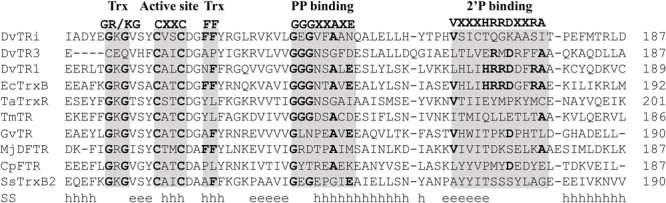
Amino acid sequence alignment of DvTRi and other low molecular weight flavin-containing TRs. The alignment was performed by the use of PROMALS3D (Pei et al., 2008). Abbreviation (organism, enzyme, NCBI accession number): DvTRi (*Desulfovibrio vulgaris* Hildenborough, non-NADP(H) TR, YP_010676.1); DvTR1 (*Desulfovibrio vulgaris* Hildenborough, NTR, YP_011055.1); DvTR3 (*Desulfovibrio vulgaris* Hildenborough, NADH-TR, YP_009601.1); EcTrxB (*Escherichia coli*, NTR, WP_001460710); TaTrxR (*Thermoplasma acidophilum*, non-NAD(P)H TR, WP_010901395, 3CTY); TmTR (*Thermotoga maritima*, NADH-TR, AAD35951.1); GvDTR (*Gloeobacter violaceus*, Deeply rooted bacterial non-NAD(P)H TR); Mj-DFTR (*Methanocaldococcus jannaschii*, Deazaflavin-dependent Flavin-containing TR, Q58931); CpFTR (*Clostridium pasteurianum*, Ferredoxin-TR, WP_015617437.1); Ss-TrxB2 (*Sulfolobus solfataricus*, putative non-NADPH TR, WP_010923960.1). SS, consensus predicted secondary structures; h, alpha helix; e, beta strand. Gray bars: conserved motifs and residues as found in L-NTRs. Trx, thioredoxin binding; PP, pyrophosphate group of NAD(P)H. 2′P, phosphate group of NADPH.

The lack of reactivity of DvTRi with DvTrx1 (Pieulle et al., 2011) was surprising given the fact that hot-spot residues that make contacts across the interface between conventional TRs and Trxs are well conserved in the DvTRi sequence (**Figure 1**), (Gustafsson et al., 2007). On the contrary, for DvTrx3, the result was expected because these residues are not conserved in DvTR3 (**Figure 1**), probably involving a strict specificity for DvTrx3. On the other hand, the classic Arg-rich NADPH binding motif VxxxHRRDxxRA is not conserved in DvTRi (**Figure 1**).

To test the ability of NAD(P)H to reduce FAD in DvTRi, the enzyme was firstly incubated with either NADPH or NADH and reduction of the FAD cofactor through changes in the visible part of the spectra was followed. As expected, incubation of DvTRi with NAD(P)H, over 60 min, did not lead to any significant reduction of FAD as indicated by the small change in the shoulder at 460 nm (Supplementary Figures S3A,B). The increase of the concentration of NAD(P)H until 1 mM did not allow the reduction of the FAD (data not shown). As a control, a rapid reduction of the bound FAD represented by a dramatic decrease in the absorbance, was observed when an excess of dithionite was added. It should be noted that the absence of activity with NAD(P)H was not due to an immature form of the recombinant enzyme since the ratio of FAD to subunit of DvTRi was 0.84. As previously demonstrated (Pieulle et al., 2011), we found that DvTR1 and DvTR3 were efficiently reduced by their specific reductants (Supplementary Figures S3C,D).

We also used the sensitive and convenient enzymatic assay described by Yang and Ma (2010) to measure the NAD(P)H-dependent reduction of benzyl viologen by the FAD group of the TRs. In this way, the benzyl viologen oxidoreductase (BVOR) activity of DvTRs in the presence of 1 mM NADPH or NADH was evaluated. While BVOR activities of 0.5 and 2.7 U/mg were found for DvTR1 and DvTR3, respectively, no BVOR activity was detected for DvTRi. These results confirmed that neither NADPH nor NADH are able to reduce the FAD of DvTRi.

### DvTRi Is the Redox Partner of DvTrx1

**FIGURE 2:**
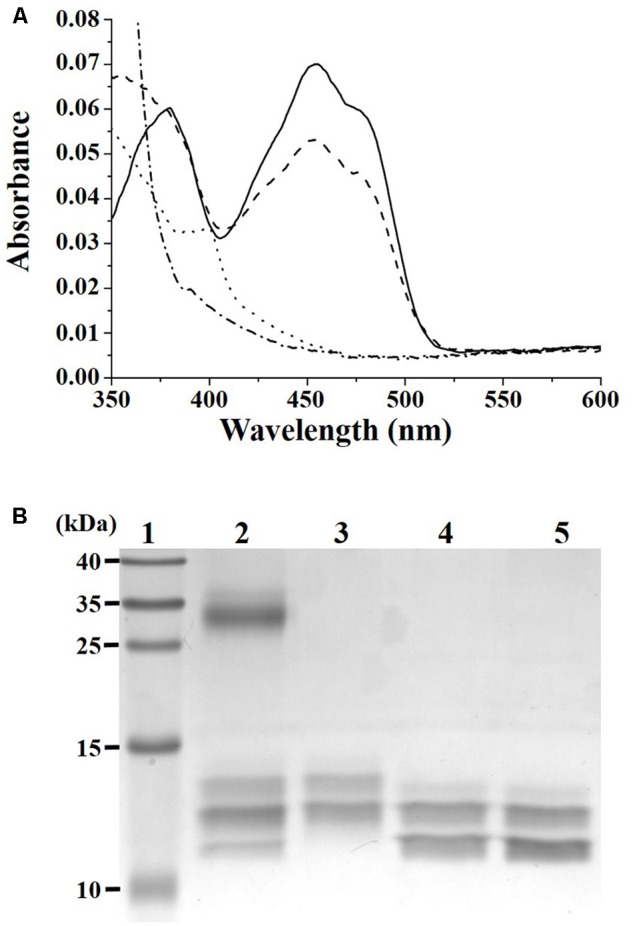
**(A)** Demonstration of DvTRi/DvTrx1 reactivity. Reduction of DvTRi was achieved by use of a modified xanthine/xanthine oxidase system as described in the Materials and Methods section. Oxidized DvTRi (solid line) was reduced with xantine as the source of electrons over the course of 60 min, in order to obtain a stable reduced form of the enzyme (dotted line). Upon addition of DvTrx1, the sample was reoxidized within 30 s (dashed line). As a control, oxidized DvTRi was reduced with dithionite (dashed-dotted line). **(B)** Gel-based oxidation states of DvTrx1. Lane 1: Molecular weight standards; Lane 2: 2 equivalents of DvTrx1 added to DvTRi (previously reduced by the xantine/xanthine oxidase reduction system) and then incubated with a thiol reactive probe (AMS); Lane 3: DvTrx1 reduced by 1 mM DTT (10 min at 25°C) and then incubated with AMS; Lane 4: DvTrx1 as prep plus AMS; Lane 5: DvTrx1 added to the xantine/xanthine oxidase system without DvTRi and then incubated with AMS.

To verify that DvTRi can catalyze a disulfide exchange with DvTrx1, oxidized DvTRi was pre-reduced using the xanthine/xanthine oxidase system. The spectrum of DvTRi reduced with xanthine was similar to the spectrum recorded after the addition of an excess of dithionite to the oxidized enzyme (**Figure 2A**). This result showed that a stable reduced form of DvTRi can be produced. Addition of DvTrx1 increased drastically the absorbance at 460 nm, which corresponds to the oxidation of the FAD and suggests that DvTRi catalyzes a disulfide exchange with DvTrx1 leading to the reduction of the disulfide bond of DvTrx1 (**Figure 2A**). The same experiment was performed with DvTrx3 and as expected no change was observed in the absorbance at 460 nm (data not shown).

Reduction of DvTrx1 by DvTRi was then tested by electrophoretic mobility change. Two bands were observed on the gel for the oxidized form of DvTrx1 (**Figure 2B**, lane 4). The same pattern was obtained when DvTrx1 was incubated with the xanthine/xanthine oxidase system before AMS was added (**Figure 2B**, lane 5). These results indicated that, as expected, the xanthine/xanthine oxidase system was not able to reduce the disulfide bond of DvTrx1. On the contrary, when DvTrx1 was reduced by DTT before AMS was added, the band pattern changed noticeably: the higher electrophoretic mobility band disappeared and one band with a lower mobility appeared (**Figure 2B**, lane 3). We concluded that this band pattern corresponded to the reduced form of DvTrx1. The two same bands were observed when DvTrx1 was incubated with DvTRi pre-reduced by the xanthine/xanthine oxidase system (**Figure 2B**, lane 2) strongly suggesting that DvTRi was able to reduce the disulfide bond of DvTrx1. In lane 2, the presence of a third band with a higher mobility indicated that Trx1 was not totally reduced under our conditions.

**FIGURE 3:**
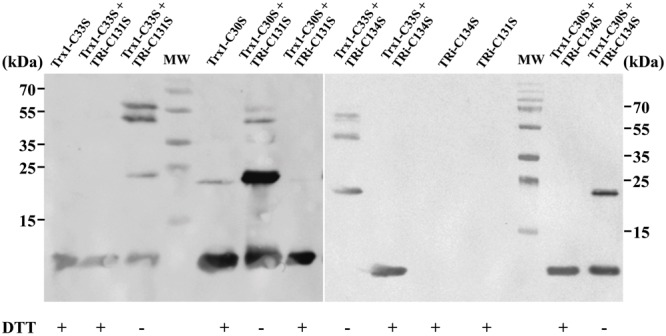
Immunodetection of DvTRi-DvTrx1 complex after non-reducing SDS-PAGE. After the complexation reaction, protein samples were resuspended in a loading buffer containing or not DTT and separated on a SDS PAGE. MW: molecular weight standards.

To confirm the direct interaction of DvTRi with DvTrx1, four cysteine variants of DvTRi and DvTrx1 were generated: TRi-C131S, TRi-C134S, Trx1-C30S and Trx1-C33S. We hypothesized that in these variants, a single active cysteine-thiol (in either protein) will lead to the arrest of the reaction and the production of an intermediate (DvTRi/DvTrx1 complex) where both proteins are covalently attached *via* a disulfide bond. The four possible combinations were tested and the presence of the complex was confirmed by gel electrophoresis under non-reducing conditions followed by western-blot using antibodies against EcTrxA. One band corresponding to the molecular weight of the complex DvTRi-DvTrx1 (∼47 kDa) was detected in all combinations except for the combination TRi-C134S and Trx1-C30S (**Figure 3**, lanes 3, 6, 8, and 14). This band was absent when the sample was reduced by DTT. A slower-migrating band (∼60 kDa) was also detected but was absent under reducing conditions and in the two controls Trx. This band should also correspond to the covalent complex with an unexpected decrease of the electrophoretic mobility. Note that the Trx1 variants can form a homodimer (molecular weight ∼24 kDa) under non-reducing conditions (**Figure 3**, lanes 3, 5, 6, 8, and 14). These results demonstrated that mixed disulfides can form from three combinations of variants and confirmed that DvTRi and DvTrx1 interact *in vitro*. Moreover, the higher yield of formation was observed for TRi-C131S-Trx1-C33S. This supports the conclusion that Cys134 of DvTRi would be the one that initiates the dithiol-disulfide interchange with DvTrx1. In EcTrxB, it has been demonstrated that the interchange thiols cysteine is also the second cysteine of the active site (Veine et al., 1998).

All together, these data demonstrate that DvTRi reduces DvTrx1 *in vitro* and suggest that, in *Dv*H, the conventional TR1 and the atypical TRi are both able to reduce DvTrx1 with a distinct electron source.

### DvTRi Plays a Role against Disulfide Stress

**FIGURE 4:**
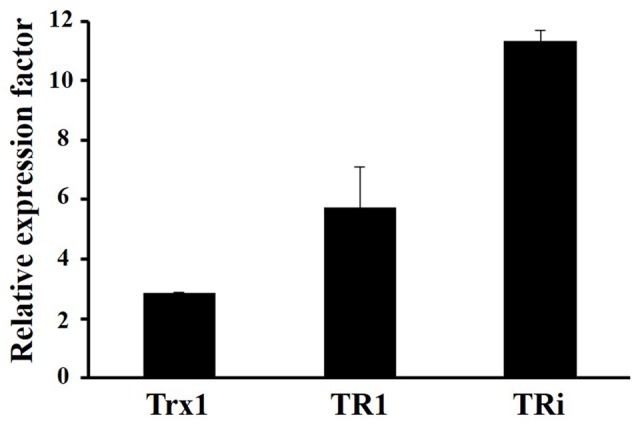
Transcriptional analyses of *Dv*H Trx systems in oxidative conditions. Data were obtained from 3 independent-cultures of WT strain exposed to air or kept in anaerobiosis during 1 h.

A *tri* gene deletion mutant was constructed to gain information on the functional role of DvTRi in *Dv*H. The deletion of the *tri* gene did not significantly affect the growth of the cells in rich and minimal lactate/sulfate media (Supplementary Figure S4). Sensitivity to diamide was used to establish if DvTRi was important for thiol/disulfide homeostasis. Indeed, diamide is a thiol-oxidizing agent that mimics disulfide stress due to oxygen exposure in anaerobic bacteria (Reott et al., 2009). The WT strain was compared to the Δ*tri* mutant strain in diamide disk diffusion assays with a defined minimal medium. Increased sensitivity was observed in the Δ*tri* mutant as indicated by a higher inhibitory zone around the diamide-containing disk for this mutant compared to the WT strain (**Table 1**). The diamide susceptibility phenotype of the mutant was successfully complemented with the *tri* gene (strain Δ*tri*/pG6*tri*) (**Table 1**). These results indicate that when the environmental conditions are too oxidative, DvTRi participates to the control of the thiol/disulfide homeostasis in *Dv*H, probably by regenerating oxidized DvTrx1. This functional role is supported by transcriptional analyses showing that the *tri* gene was induced ∼11-fold during air exposure whereas the expression of *tr1* and *trx1* genes were increased by of ∼5.8-fold and ∼2.9-fold, respectively (**Figure 4**).

**Table 1:** Influence of DvTRi on diamide resistance of *Desulfovibrio vulgaris.*

Strain	Zone of inhibition^1^	
	1 M	1.5 M
WT/pBMG6	43 ± 3	50 ± 0.5
Δ*tri*/pBMG6	51 ± 3	60 ± 1
Δ*tri*/pG6*tri*	43 ± 2	50 ± 1

*^1^The values are mean diameters (in millimeters) of growth inhibition zones measured in two independent experiments performed in triplicate. Twenty microliters of a 1 or 1.5 M diamide solution was applied to a filter placed in the center of each plate containing defined minimal medium.*

### Crystal Structure of DvTRi

**FIGURE 5:**
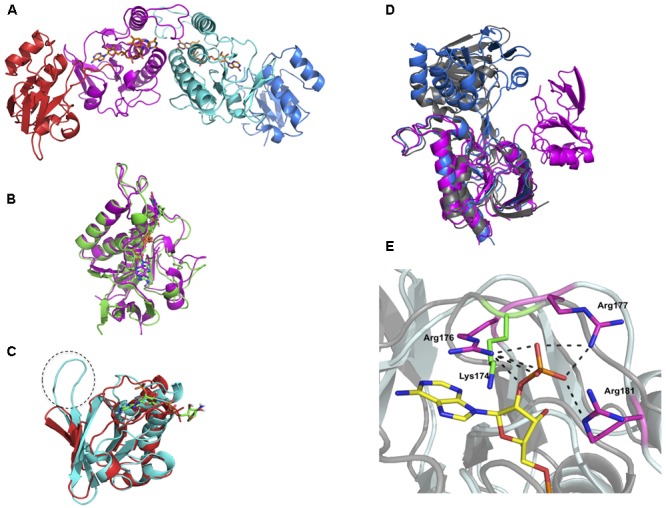
Crystal structure of DvTRi. **(A)** Homodimer of DvTRi (red and blue: pseudo NADPH-domains, magenta and cyan: FAD-domains); **(B)** Superposition of DvTRi (magenta) and EcTrxB (green) FAD-domains; **(C)** Superposition of DvTRi (red) and EcTrxB (cyan) NADPH-domains; **(D)** Superposition of EcTrxB FO (gray, PDB code: 1TDF) and EcTrxB FR (blue), PDB code: 1F6M) forms with TRi (magenta). **(E)** Superposition of NADPH-binding site of EcTrxB in pale cyan (PDB code: 1TDF) with DvTRi in gray. NADPH analog is depicted in yellow. The three arginine residues present in EcTrxB are depicted in magenta and Lysine 174 present in DvTRi is depicted in green.

The crystal structure of oxidized DvTRi was solved at 2.0 Å resolution, with one physiological homodimer per asymmetric unit. Each monomer is composed of a FAD-binding domain (residues 1–109 and 231–309) and a pseudo NADPH-binding domain (residues 119–225) (**Figure 5A**) linked by an interdomain hinge region (residues 110–118 and 226–230). The structural alignment of these domains with the equivalent domains in EcTrxB (PDB ID: 1TDF) showed that the overall structures are quite similar with rmsd values of 1.39 and 1.9 Å for the FAD- and pseudo/NADPH-binding domains, respectively (**Figures 5B,C**). The FAD protein environment is well conserved in DvTRi with a similar binding mode, through a network of supramolecular interaction involving structural water molecules and the conserved binding-motifs **G**x**G**xx**G, ATG** and **G**x**FAAGD** (Supplementary Figure S5). The electron density of FAD is well defined with an occupancy close to 1 and the isoalloxazine ring of FAD is planar. We also observed that the loop from residues 223 to 231 present in EcTrxB is missing (**Figure 5C**). In the pseudo NADPH-binding domain, the signature motifs **V**xxx**HRRD**xx**RA** and **GGG**xx**A**x**E** required for NADPH binding are replaced with the **V**xxx**TQGK**xx**SI** and the **GEG**xx**A**x**N** motifs, respectively. The only basic amino acid in the motif is lysine 174, which is well superposed with arginine 176 in EcTrxB. However, it is not sufficient to stabilize the phosphate group of NADPH since its binding requires seven salt bridges with three arginines in EcTrxR (**Figure 5E**). The absence of crucial residues in DvTRi required for the binding of NADPH excludes the possibility for the enzyme to physiologically use this reductant, which is in complete agreement with our biochemical data. Two different crystallization conditions were tested and several crystals were analyzed. Whatever the crystallization conditions were, the different structures we solved from crystals 1 or 2 showed an unexpected unique conformation of DvTRi, that differs from both FO and FR conformations observed in EcTrxB structures (**Figure 5D**). The structure revealed that the two active-site cysteines (the **C**xx**C** motif) are far away from FAD (**Figure 5D**). Therefore, the enzyme must undergo a complete reorganization to bring the active site close to the FAD and several conformations would exist in solution along the reaction mechanism.

### Occurrence of TRi in Prokaryotes and Specific Features Associated to TRi Group

We first addressed the general relevance of TR proteins, through a large-scale *in silico* analysis of PNDO (*Pyridine Nucleotide-Disulfide Oxidoreductase*) proteins on all complete prokaryotic genomes available at that time. Indeed, experimentally well-characterized TRs (see Supplemental Material) used as seeds included 5 proteins (∼300 amino acid length) that solely contained a PNDO (Pyr_redox2, PF07992 in Pfam database) domain. The PNDO domain comprises FAD-binding, the active site and the NADPH-binding regions of TRs. By using this functional domain as query, we identified PNDO proteins within complete prokaryotic genomes, which constitute the primary dataset used in this study. Details on the *in silico* procedure and analyses are found in the SM.

Among the functional features of the classical TRs (e.g., DvTR1 and EcTrxB), the active site and the NADPH binding regions encompassed four conserved elements (see **Figure 1**): the Trx (GR/KG and FF) binding sites, the CxxC active site, as well as the GGGxxAxE and VxxxHRRDxxRA motifs for the binding of pyrophosphate (PP) group and phosphate (2′P) group of NADPH. As shown in **Figure 1**, GR/KG and FF residues are not conserved in DvTR3, which is in accordance with the strict specificity of this TR for the non-canonical Trx3 (Pieulle et al., 2011; Garcin et al., 2012). Similar observations can be made with less conserved residues within the PP binding sites of CpFTR or MjDFTR which are known to use ferredoxin and coenzyme F420 as electron source, respectively (Hammel et al., 1983; Susanti et al., 2016). In addition, except for DvTR1 and EcTrxB, the 2′P binding HRR residues were absent in other TRs. All of these observations clearly suggested that the active site and NAD(P)H binding regions can be used as a pattern marker for clustering and identification of specific TR groups. As mentioned in the Experimental procedures section, the iterative searches found 17, 3171, and 26 homologs for TRi, TR1/FdR and TR3 groups, respectively (Supplementary Figure S6). Interestingly, EcTrxB proteins that did not previously show similarity network relationships (see SM) with DvTR1, were found in TR1 group of homologs after iterative searches (Supplementary Figure S6), which demonstrates the robustness and sensitivity of the procedure. In the final step, DvTR1 homologs without the two conserved cysteines within the active site regions were considered as ferredoxin (flavodoxin) reductase (FdR, Chen et al., 2015) proteins (639 proteins in total) and were removed from the set of specific TR1 group of homologs. The TR3 set of homologs was split in two distinct subgroups: TR3 and degenerated *Clostridium* TR1 (dcTR1) subgroups with 8 and 18 homologs, respectively. This division is due to the absence of the non-canonical Trx3 partner (Pieulle et al., 2011) within the clostridial genomes. The 17 TRi homologs were found in the Deltaproteobacteria class (mostly in Desulfovibrionales order, e.g., *Desulfovibrio vulgaris* DP4) as well as in the Clostridia class (mainly in Clostridiales order, e.g., *Clostridium thermocellum* ATCC 27405) (Supplementary Table S3). Except for *Ruminoclostridium* species, organisms containing TRi also harbor TR1 (Supplementary Table S3; see also right part in **Figure 7** downstream).

**FIGURE 6:**
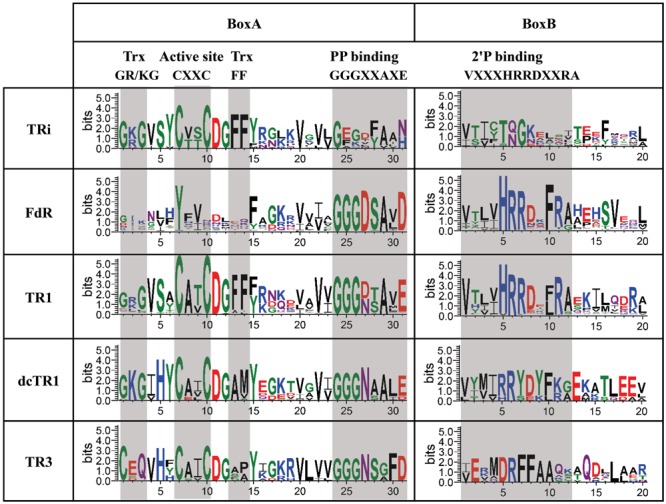
Sequence logos of “Reduced Active Site” region of TRi, FdR, TR1, dcTR1 and TR3 subgroups. Conserved elements are clustered in two boxes (BoxA and BoxB): Trx (thioredoxin) binding sites, Active site, PP binding (Pyrophosphate_NADPH_-binding) and 2′P binding (2′-phosphate-binding) motifs. These elements are shaded in light gray.

This analysis allowed us to define specific features associated with TRi, FdR, TR1, dcTR1 and TR3 proteins. As expected, the first 23 amino acids of the FdR BoxA region completely differed from the ones of the TR1 type, therefore confirming in a large-scale analysis that their active site motifs diverge even with a similar NADPH binding motif (**Figure 6**). The active site regions of TRi, TR1, dcTR1 and TR3 homologs are quite similar, except for the Trx GR/KG positions (CxQ in TR3) and the Trx F positions (mainly M and P amino acid in dcTR1 and TR3, respectively). Between the Trx GR/KG and the CxxC motif, the VSY residues (position 4–6 in sequence logo) are well conserved in TRi and TR1 while the serine residue is replaced by histidine in TR3 and dcTR1. The PP binding motifs (GGGxxAxE, position 24–31) were found highly conserved in TR1 and TR3, but highly degenerate in TRi. This motif was demonstrated to interact with one phosphate group through the backbone atoms (Kleiger and Eisenberg, 2002; Hua et al., 2014), as well as with the third glycine known to perform a close contact of the N-terminus of the α-helix to the pyrophosphate of the nucleotide (Wierenga et al., 1986). In addition, the 2′P binding motif remains clearly different between TR1/FdR and TRi/dcTR1/TR3 group of homologs (**Figure 6**), suggesting that this region is specific to each TR group and can be used to discriminate the members of these groups. In particular, the strict conservation of the GKG sequence involved in the canonical Trx-binding and as well as a partial conservation of the 2′P binding motif for NADPH, also contributed to define and characterize the novel dcTR1 group. Altogether, our large-scale study allowed to define L-TRs specific signatures: the highly conserved GGGxxAxE and VxxxHRRDx[F/L]R motifs for NADPH binding regions, and the G[R/K]G motif and the Phe residue for Trx binding sites.

### Evolutionary History of DvTRs

**FIGURE 7:**
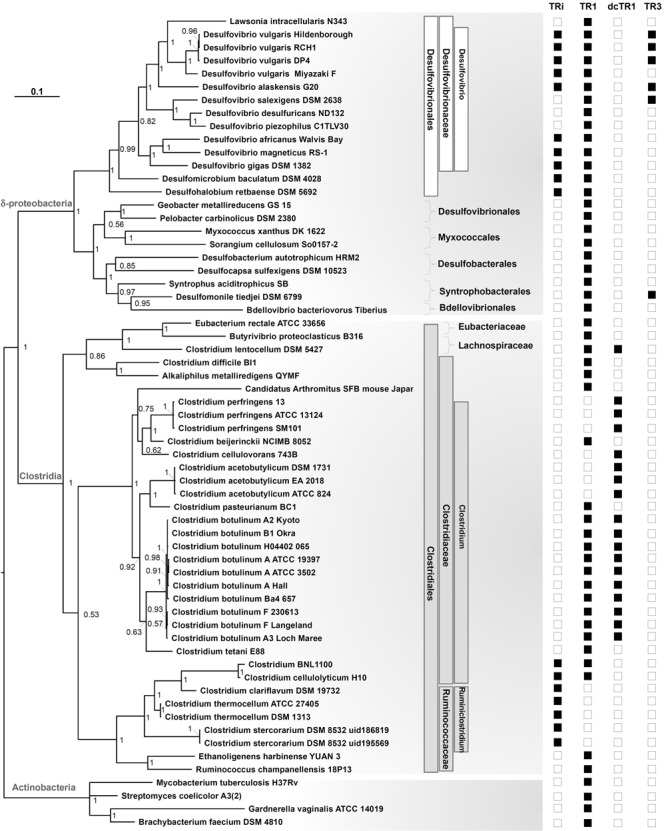
Phylogenetic tree of the 16S rRNA gene in Clostridia and Deltaproteobacteria. Data included all organisms containing TRi, and dcTR1/TR3 homologs, in addition to TR1 proteins when present or in selected organisms. Sampling also included 16s rRNA sequences from Actinobacteria as an outgroup. The tree made through a Bayesian 50% majority rule consensus tree, with associated branch lengths. Values on nodes refer to posterior probabilities (PP). For each species, the presence of TR homologs is signed by black squares and their absence signed by blank squares. Taxonomic lineages (Order, Family, Genus) were shown in white and gray boxes for Deltaproteobacteria and Clostridia classes, respectively.

**FIGURE 8:**
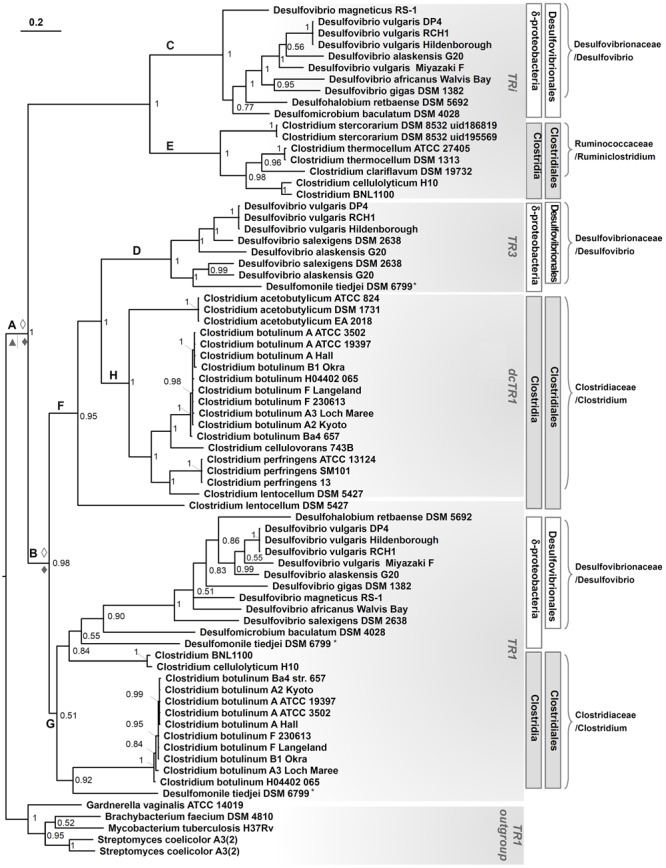
Phylogenetic tree of TRi, TR1, dcTR1 and TR3 proteins. Data included all organisms containing TRi, and dcTR1/TR3 homologs, in addition to TR1 proteins when present or in selected organisms. Sampling also included TR1 protein sequences from Actinobacteria as an outgroup. The tree made through a Bayesian 50% majority rule consensus tree, with associated branch-lengths. Values on nodes refer to posterior probabilities (PP). The organism marked with a “star” is belong to Deltaproteobacteria group. ◊, Duplication in Desulfovibrionales strains; ♦, Duplication in Clostridiales strains, ▲, Speciation in Clostridiales strains.

To shed light on the origin and evolution of DvTRs, phylogenetic trees representing the distribution of TRs over the prokaryotic phyla, classes, orders, families and genera were fulfilled (see Supplementary Figure S7 and **Figure 7**). In addition, the evolutionary events that have occurred over time were figured out (**Figure 8**). We inferred the presence of a TR in the ancestor of a phylum if we found a monophyletic group of TRs that corresponds to the phylum and if the corresponding genes are well distributed within the phylum. As TR1 genes are found in all prokaryotic phyla (Supplementary Figure S7), one can reasonably assume that TR1 was present (originated from) in the LBCA (Last Bacterial Common Ancestor) and LACA (Last Archaeal Common Ancestor) before its propagation in prokaryotic phyla, through vertical inheritance, duplication or HGT events. The presence of TRi, dcTR1 and TR3 within a limited number of taxonomic classes (here Clostridia and Deltaproteobacteria) suggested that their acquisition arose late in the evolution, probably in specific taxonomic families (e.g., *Desulfovibrionaceae*) through HGT or duplication events. On the contrary, the absence of TRi and TR3 within Archaea strongly suggested that the LACA had neither TRi nor TR3.

To determine if the groups (clades) recovered in the TR phylogenetic tree reflect the organismal phylogeny, we estimated a phylogeny for the Deltaproteobacteria, Clostridia classes and related taxonomic groups using 16S rRNA gene sequences (**Figure 7**). The overall topology of the TR tree was fitted with the 16S rRNA tree, except for *Desulfomonile tiedjei* DSM 6799 (a Deltaproteobacterial organism) and Clostridium BNL 1100/*Clostridium cellulolyticum* H10. For *Desulfomonile tiedjei* DSM 6799, the second TR1 copy emerged within the Clostridia taxonomic class while for *Clostridium* BNL 1100/*Clostridium cellulolyticum* H10 species, TR1 copies are closely related to *Desulfovibrio* strains (**Figure 8**). These suggest that HGT events of TR1 sequences had occurred between *Clostridium* and *Desulfovibrio* species.

The TR phylogenetic tree showed several monophyletic groups corresponding to the Clostridia or Deltaproteobacteria classes, most of them are well supported (with posterior probability > 0.5). Interestingly, four major and distinct subgroups, each associated to TRi, TR1, dcTR1 or TR3, were clearly separated within the tree, therefore suggesting a distinct evolutionary history for each type of TR and probably distinct molecular functions. In the case of TRi and dcTR1/TR3, they probably originated from TR1 through duplications in *Clostridium, Lachnospiraceae, Desulfovibrio*, and *Syntrophobacterlaes* genera. Indeed, in *Desulfovibrionales*, a possible evolutionary history may have been a duplication of TR1 common ancestor (**Figure 8**, node A), followed by gene specialization to TRi through mutational changes in particular within the NADPH-binding elements (here PP and 2’P binding motifs) of the ancestral TR1. This hypothesis is supported by the fact that the majority of SRB strains (10 over 14) and some *Clostridium* (2 over 34) (see **Figure 7**) harbors both TR1 and TRi. Since the duplication in A seems to happen early in the evolution, it should be noticed that this hypothesis implies loss of TRi in some *Desulfovibrio* and *Clostridium* species (e.g., *Desulfovibrio salexigens* DSM 2638, *Clostridium botulinum* strains; **Figure 8**, nodes C and E) during time. In the case of TR3 evolutionary history, a second and recent duplication (**Figure 8**, node B) of TR1 may happen to produce a copy of TR1 that was subjected to acid amino changes to become TR3 (in *Desulfovibrio* species, **Figure 8**, node D) and dcTR1 (in *Clostridium* species, **Figure 8**, node H). This evolutionary hypothesis is supported by the fact that (i) TR3/dcTR1 (node F) and TR1 (node G) share a common node (node B) for *Clostridium* and *Desulfovibrio* species; (ii) a number of organisms (6 organisms from Deltaproteobacteria, 11 organisms from Clostridia) contains both TR1 and TR3/dcTR1. Altogether, these distinct evolutionary histories between TRi, TR1, dcTR1 and TR3 through duplications events may have led to functional divergence (or subfunctionalization or specialization) of the duplicated proteins (here TRi and TR3/dcTR1) over the evolutionary time. For instance, while TR1, the ancestral gene, may have kept and performed the standard function, the paralogous genes (TRi and TR3/dcTR1), were adapted to particular and specific functions under specific environmental and growth conditions.

## Discussion

Previous studies of the thiol redox systems of the strict anaerobe, *Dv*H, have revealed new types of Trx and TR. In addition to the canonical Trx/TR system, *Dv*H contains two others TR systems with still unknown functions, i.e., a TR3/Trx3 system and an isolated TR (DvTRi). We have previously shown that DvTRi was unable to reduce the conventional DvTrx1 or the atypical DvTrx3 in the presence of NADPH (Pieulle et al., 2011). In the present study, analysis of the amino-acid sequence of DvTRi showed the presence of the main elements for the recognition of the conventional Trx. DvTRi reduced by a xanthine/xanthine oxidase system was found to react and form a complex with DvTrx1 but not with DvTrx3. The observation that DvTRi does not react with DvTrx3 is in agreement with the strict recognition of DvTrx3 by DvTR3 probably due to the positive surface around the active site of DvTrx3 compared to the highly conserved hydrophobic patch found in canonical Trxs as EcTrxA (Garcin et al., 2012). These data led us to propose that, in addition to DvTR1, DvTRi would be able to regenerate the pool of reduced Trx1 in *Dv*H. It is noteworthy that, to our knowledge, *Dv*H is the first example of prokaryotic cells where two TRs are able to reduce the same Trx. The opposite situation was found in the anaerobe *Bacteroides fragilis* with one TR for six Trxs or the strictly anaerobic methane-producing archaea *Methanosarcina acetivorans* that contains a single TR and seven Trx homologs (Reott et al., 2009; McCarver et al., 2017). We also confirm here that NADH and NADPH are not able to reduce the FAD of DvTRi, allowing us to definitely conclude that these reductants do not react with this enzyme. Two TRs dedicated to the same Trx are likely to be related to the use of distinct electron source. Study of the structural properties of DvTRi support this notion. Even if an overall fold similar to that of the NADPH-dependent TRs (NTR) was found for both the FAD-binding domain and the pseudo NADPH-binding domain, especially with a high similarity between the FAD-binding domain and the Trx interface region with those of NTRs, major differences were also noted. The reductant-binding pocket of TRi does not appear to be designed to bind NADPH, with the absence of hot spot residues for binding the 2’phosphate group of the coenzyme. The lack of activity with NADPH has been already described for the TR of an acidophilic archaeon (TaTrxR) (Hernandez et al., 2008). Similarly, a recent report demonstrates that a TR (GvDTR) from an ancient aerobic cyanobacterium (a close structural homolog of NTR) is also unable to use NADPH as reductant (Buey et al., 2017). As in DvTRi, the motif VxxxHRRDxxRA is also absent in the amino acid sequences of the two enzymes (TaTrxR and GvDTR). In addition, the crystal structure of DvTRi revealed another characteristic since the enzyme adopts an unexpected conformation. A similar property has been described for GvDTR (Buey et al., 2017), showing that the mechanism of the enzyme requires conformational flexibility between the FAD- and the pseudo NAD(P)H-domains (Supplementary Figure S8). Moreover, while all other structures of uncomplexed NTRs were in the FO state, the structure in the crystal of the non-NADPH TR from *T. acidophilum* was found to be in the FR form (Supplementary Figure S8) (Hernandez et al., 2008). Thus, the structural conformations observed in several crystal structures of TRs that are not NADPH-dependent suggest a higher flexibility of these enzymes compared to standard NTRs, and several conformations might occur during the reaction. Unfortunately, based on structural analyses, the major conformational differences between NADPH-independent TRs do not allow for the determination of the electron donors used by DvTRi. *In vivo* and *in vitro* investigations are therefore necessary to clearly identify the nature of the reductant. This is a great challenge as the most obvious candidates, NADPH and NADH, are excluded by this study. Moreover, very recently, a novel F420-dependent TR (*Mj-*DFTR) has been discovered in the methanogenic archaeon, *Methanocaldococcus jannaschii* (Susanti et al., 2016). However, the exploration of the distribution of the genes encoding the F420 biosynthesis enzymes in the public genomes revealed that these genes are not present in *Desulfovibrio* species (Ney et al., 2017) allowing us to conclude that F420 is not the electron source of DvTRi. In the eighties, an interesting study showed that *Clostridium pasteurianum*, a strict anaerobic bacterium, uses a Fe_4_S_4_-type Fd to reduce Trx *via* a flavoenzyme (Hammel et al., 1983). The sequence of the clostridial TR displays no conservation of the NADPH-binding residues of TRs and no significant sequence similarities with the reductant-binding pocket of DvTRi indicating that DvTRi reductant should not be a ferredoxin. The flavodoxin, a small flavoprotein that contains one FMN and that functions as electron carrier in reactions of low oxidation-reduction potential (O’Farrel et al., 1998) could be a candidate as reductant of DvTRi.

In this context, the function of DvTRi in *Dv*H has been addressed by a classical gene deletion approach. The *tri* deletion mutant did not exhibit a growth phenotype in lactate/sulfate medium even in a minimal growth medium that contains no exogenous free thiol source. This result is in contrast with the incapacity of the *trxB* deletion mutant of another strict anaerobe, *Bacteroides fragilis*, to grow in the absence of cysteine or dithiothreitol (Rocha et al., 2007) and demonstrates that the NADPH/TR1/Trx1 system is sufficient to manage the redox homeostasis of the cytoplasm under anaerobic conditions. However, a *tri* deletion mutant is found to be more sensitive than the parent strain to the effects of diamide that mimic disulfide stress induced by oxygen. Furthermore, the *tri* gene is highly upregulated when *Dv*H cells are exposed to air, which is in agreement with genome-wide transcription analyses designated to identify novel genes that respond to oxidative stress in *Dv*H (Zhang et al., 2006; Mukhopadhyay et al., 2007; Pereira et al., 2008; Zhou et al., 2010). We can thus propose that DvTRi is an asset to combat oxidative damage in *Dv*H cells, designed to aid TR1 in reducing the pool of oxidized Trx1 highly requested during the stress period with the use of another electron source than NADPH. The identification of the physiological electron donor of DvTRi will clarify how DvTRi supports the NTR system in aerotolerant strict anaerobes.

Through *in silico* approaches, functional and phylogenomics analyses of TRi homologs were performed. All TRi homologs contain the three residues (Lys or Arg residue and two Phe residues) responsible for most of L-TR-Trx interface stability (Obiero and Sanders, 2011). On the contrary, the residues implicated in NAD(P)H-binding are partially or fully absent. However, in the 2’P group-binding region, only few residues are well conserved among TRi proteins with a TQ/NGK motif instead of the HRRD motif. Like in *Dv*H, we can hypothesize that in SRBs and Firmicutes which contain TR1 and TRi homologs, the substitutions selected during the evolutionary history of TRi excluded NAD(P)H as substrate and therefore improved the capacities of the Trx system. As three thermophilic cellulolytic clostridia harbor only TRi homologs, this enzyme must play the essential role of the Trx system and we can postulate that in these *Clostridium* species the electron donor is the same as the one of DvTRi. Evolutionary studies showed that TRi, TR1, dcTR1 and TR3 underwent a complex evolutionary history (with TR1 as a common gene ancestor in Archaea and Bacteria). These were affected by both gene duplications and horizontal gene transfer events, leading to the appearance of TR3/dcTR1 and TRi through subfunctionalization over the evolutionary time. A recent *in silico* identification of deazaflavin-dependent flavin-containing thioredoxin reductase (DFTR) homologs (e.g., Mj-DFTR, from *Methanocaldococcus jannaschii*, Susanti et al., 2016) within *nr* database showed that these proteins were limited to the deeply-rooted methanogens (in Euryarchaeota phylum), therefore suggesting that these atypical TRs (TRi, TR3, dcTR1 and Mj-DFTR) are only found in particular taxonomic clades and should be useful in specific environmental niches. Multiple alignment of DvTRi with non-NADPH dependent TRs shows that there is a high variability in the region corresponding to the 2′P binding sequence that probably reflects the adaptation of TRs for the use of distinct source of electrons (**Figure 1**). These mutations should be the cause or the consequence of the specific metabolic and/or environmental conditions related to the microorganisms having non-NADPH dependent TR.

## Author Contributions

LP, CC, and ET conceived and designed the experiments. OV, TT, CC, GB, EC, and LP performed the experiments. OV, TT, CC, GB, EC, ET, and LP analyzed the data. TT, CC, AD, ET, and LP wrote the paper. All authors read and approved the final manuscript.

## Conflict of Interest Statement

The authors declare that the research was conducted in the absence of any commercial or financial relationships that could be construed as a potential conflict of interest.

## Footnotes

1 ftp:/ftp.ncbi.nih.gov/genomes/

2 http://www.ncbi.nlm.nih.gov/genome/browse/representative/

3 http://tree.bio.ed.ac.uk/software/figtree/

## Abbreviations

AMS: 4-acetamido-4′-maleimidylstilbene-2,2′-disulfonic acid
DTT: dithiothreitol
*Dv*H: *Desulfovibrio vulgaris* Hildenborough
Fd: Ferredoxin
TR: thioredoxin reductase
Trx: thioredoxin
