# Supplementary material for: Biochemical Function, Molecular Structure and Evolution of an Atypical Thioredoxin Reductase from Desulfovibrio vulgaris
Source: Front Microbiol. 2017 Sep 29;8:1855. doi: 10.3389/fmicb.2017.01855 (PMC5627308; doi:10.3389/fmicb.2017.01855)
Supplement: Supplementary file 1 [file Data_Sheet_1.pdf]

# *Supplementary Material*

# Biochemical function, molecular structure and evolution of an atypical thioredoxin reductase from *Desulfovibrio vulgaris*

**Odile Valette[1,*], Tam Thanh Thi Tran[1,*], Christine Cavazza[2,3,4], Elodie Caudeville[1], Gaël Brasseur[1], Alain Dolla[1], Emmanuel Talla[1§], and Laetitia Pieulle[1§]**

[§]**Correspondence**: L. Pieulle: pieulle@imm.cnrs.fr and E. Talla: talla@imm.cnrs.fr.

[1]Aix Marseille Univ, CNRS, LCB, Marseille, France.
[2]Univ. Grenoble-Alpes, Laboratoire de Chimie et Biologie des Métaux, F-38054 Grenoble, France.
[3]CNRS, LCBM, UMR 5249, F-38054 Grenoble, France.
[4]CEA-Grenoble, DRF/BIG/CBM, F-38054 Grenoble, France.

*These two authors contributed equally to this work

**Keywords:** Thioredoxin reductase, Oxidative stress, Crystal structure, Phylogenomics, *Desulfovibrio*, Anaerobes.

**Running title**: *An atypical thioredoxin reductase in Desulfovibrio*

## Supplementary Methods

*Datasets*

The complete genomes (including their taxonomy lineages) of 2772 prokaryotic (2607 bacterial and 165 archaeal) organisms available in March 2015 were downloaded from NCBI ftp site (ftp:/ftp.ncbi.nih.gov/genomes/) and constituted the primary data source. This data was cross-compared with NCBI representative prokaryotic genomes (http://www.ncbi.nlm.nih.gov/genome/browse/representative/) leading to 1084 representative complete genomes. Experimentally-known thioredoxin reductase (TR) proteins used as reference seed proteins are presented in **SuppTable 1**.

*Identification of pyridine nucleotide-disulphide oxidoreductase proteins*

The HMMER-3 package (Mistry et al., 2013) (Mistry et al., 2013) and self-written Perl scripts were then used to search for protein having a pyridine nucleotide-disulphide oxidoreductase (PNDO, PF07992 from Pfam 28.0 database ((Finn et al., 2016), 292 amino acid positions) domain within representative and all complete genomes (see **Fig. S2**). Since seed proteins (*e.g.* DvTRi) (**SuppTable 1**) are solely composed of a PNDO domain, the presence of this functional domain was the main requisite. Alignments with (i) a score higher than the Pfam trusted thresholds and (ii) a length between than 263-350 amino acids (corresponding to 0.9-1.2 fold of the PNDO profile length) were considered significant, leading to list of proteins with a PNDO domain from representative or all complete prokaryotic genomes. Indeed, this correspond to ~80% of the total number of alignments (**SuppFig. 1**) generated by the HMMER software. The obtained PNDO proteins were also analyzed with the same software in order to locate additional known functional domains, and in-house Perl scripts were subsequently used to define the domain organization.

*Reconstruction of Protein Similarity Network with PNDO proteins*

The protein similarity networks (PSNs) were constructed as described (Zhang et al., 2011). For each sequence of the list of PNDO proteins (from representative genomes) (see above), the PNDO sequence region was extracted, representing the novel sequences associated to each representative PNDO protein. With these new sequences, all pairwise alignments were performed with the "*blastall -p blastp"* program (BLAST package (Altschul et al., 1997)) without self-blast sequences. Then a series of E-value thresholds were applied for the selection of sequence pairs with significant similarity, followed by the distribution of pairwise alignments E-value in order to define the optimal E-value cut-off (**SuppFig. 2**). Finally, significant alignments (with E-value less than the optimal E-value) were used to construct the PSN. Each node in the PSN indicates a PNDO protein, and the edge indicates that two nodes share significant similarity with an E-value less than the selected cut-off. The network was visualized using Cytoscape (version 3.2.1) (Shannon et al., 2003) with the yFiles organic layout. From the PSN network, PNDO proteins with network relationships with seed proteins were defined as group of homologs associated to the reference seed proteins. Consequently, we obtained PSN group of homologs from TRi, TR1 and TR3 proteins (see **Fig. S2**) within representative genomes. Note that all members of the 3 groups solely display a PNDO domain.

## Supplementary Text

*Occurence and distribution of PNDO proteins in representative and all prokaryotic organisms*

A large-scale *in silico* identification of PNDO (*Pyridine Nucleotide-Disulphide Oxidoreductase*) proteins was performed on all complete prokaryotic genomes. The *in silico* procedure is described in the Supporting Methods section. Indeed, experimentally well-characterized TR proteins (see **SuppTable 1**) include 5 proteins (~300 amino acid length) from different phyla as Deltaproteobacteria (e.g. *Desulfovibrio vulgaris* Hildenborough), Gammaproteobacteria (e.g. *Escherichia coli* IAI39) and Euryarchaeota (e.g. *Thermoplasma acidophilum* DSM 1728) phyla. These proteins solely contain a PNDO (Pyr_redox2, PF07992 in Pfam database, HMM profile of 292 amino acid positions) domain which comprises the « active site » and « NADPH binding » regions. As DvTR1, DvTR3, DvTRi and other reference proteins harbors a pyridine nucleotide-disulphide oxidoreductase (PNDO) domain, we defined PNDO protein as a protein containing the PNDO domain. By using this functional domain as query, we detected 32795 and 13847 PNDO proteins within 2763 and 1084 organisms from all and representative complete prokaryotic genomes, respectively. These constitute the primary dataset used in this study (**SuppTable 2**).

As expected PNDO proteins were found in all taxonomic lineage, demonstrating their diversity and their importance over the bacterial and archaeal kingdoms. The PNDO proteins present in each organism ranges from 1 (e.g. *Buchnera aphidicola* JF98, *Mycoplasma haemofelis Langford* 1) to 143 (e.g. *Vibrio parahaemolyticus O1:K33* str *CDC K4557* ) with most frequency (about 99.2%) of the prokaryotic organisms having their PNDO proteins ranging from 1 to 34. However, no PNDO protein was found in minimal genomes (*Candidatus Tremblaya princeps* and *Candidatus Carsonella ruddii* species) known to be highly degenerate, compact and reduced genomes (McCutcheon and Moran, 2012). Numerous of PNDO protein domain architectures were found to harbor only one PNDO domain (98.5% in total), while few of them contained two (e.g. YP_002602461 in *Desulfobacterium autotrophicum* HRM2), three (e.g. YP_007687501 in *Thermoplasmatales archaeon* BRNA1) or four PNDO (e.g. YP_004546210 in *Desulfotomaculum ruminis* DSM 2154) domains within the same protein. Among the proteins with one PNDO domain, about

one third (35.2%) of them contains only the PNDO domain, whereas ~25.3% and ~6.1% of them are associated with the "Pyr redox dim" (pyridine nucleotide-disulphide oxidoreductase, dimerisation domain) and "Fer4_20" (dihydroprymidine dehydrogenase domain II, 4Fe-4S cluster), respectively. The remaining proteins were associated with numerous other domains such as Reductase_C, Fer4_20, Thioredoxin_3 (see **SuppTable 2**). Altogether, these demonstrate that PNDO proteins exhibit a high diversity of additional domains that may contribute to the large diversity of functions for thiol reductase proteins.

*Occurence and distribution of DvTRs homologs in representative prokaryotic organisms*

In order to classify PNDO proteins and determine TRi groups of homologs in comparison with TR1 and TR3, we reconstructed the protein similarity network associated to these proteins, using the PNDO sequence region (see Supporting Methods section). However, due to the high amount of data and the highly memory use of the Cytoscape software, subsequent analyses were mainly performed with PNDO proteins of representative genomes. The emergence of connections between putative clusters was examined with different alignment E-value cut-offs from $10^{-20}$ to $10^{-100}$. Permissive cut-offs (e.g., $10^{-20}$) collapsed all sequences into one single cluster without any outliers, whereas more stringent cut-offs (e.g., $10^{-100}$) broke the data set into small-disconnected groups (data not shown). Through the distribution of pairwise alignment numbers (**SuppFig. 2**) with decreasing E-value cut-offs ($10^{-20}$ to $10^{-100}$), an optimal E-value cut-off of $10^{-64}$ at cross-point of the two curves, which contains the majority of pairwise alignments (67%) and almost all the distinct PNDO regions (97.4%), was defined. As shown in **SuppFig. 3** and **SuppFig. 4**, the use of this optimal cut-off produces seven major cluters and numerous peripheral clusters, and most of the clusters were formed according to protein domain architecture, instead of their taxonomic relationship. As a stack example, the "Pyr redox 2 * Pyr redox dim" cluster (the biggest one in red within **SuppFig. 3**) is present in at least ten phyla (in **SuppFig. 4**). These results clearly suggest that proteins with the same domain architecture have the similar evolutionary history of the PNDO domain instead that the inheritance lineage due to their taxonomic lineage.

In particular, PNDO proteins that solely harbor a functional domain (here the PNDO domain) were scattered in different clusters. Among these groups, seed reference proteins were gathered in two distinct clusters (the smallest ones containing 28 proteins including DvTRi and the largest cluster has 1640 proteins with other reference seed proteins) (**SuppFig. 3**), spread out in several phyla (e.g. δ-proteobacteria, Firmicutes for smallest cluster and Firmicutes, Actinobacteria, Proteobacteria for the largest cluster), with the main domain architecture made by a solely PNDO domain. In addition, DvTRi, DvTR1, and DvTR3 seed proteins only showed network relationships with PNDO proteins with an unique domain (here PNDO), therefore clearly suggest that their homologs should also contain a solely PNDO domain. Protein network relationships of DvTRi, DvTR1, and DvTR3, lead to group of protein homologs from representative genomes (**SuppFig. 5**). In particular, TRi group of homologs was formed by 11 proteins (from δ-proteobacteria and Clostridia) while TR1 network group is composed of 339 proteins (e.g. from Firmicutes, Actinobacteria, δ-proteobacteria, α-proteobacteria) and TR3 is made by 24 proteins from Clostridia, δ-proteobacteria and Thermotogales. In addition, no direct relationship was observed between seed proteins (**SuppFig. 5**). All these results clearly suggest a sequence specificity between DvTRi and other seed proteins with a network group of homologs that are distinct other group of TRs. Consequently, this result enables us to find specific sequence features associated to TRi protein groups.

## List of SuppFigures

**SuppFigure 1.** Distribution of PNDO size ratios from hmmer analysis with representative prokaryotic organisms.

**SuppFigure 2.** Distribution of pairwise alignments E-values between PNDO sequence regions from representative prokaryotic organisms.

**SuppFigure 3.** Protein similarity network (PSN) reconstructed with PNDO domains from representative prokaryotic organisms in various functional domain architectures.

**SuppFigure 4.** PSN reconstructed with PNDO sequence regions from representative prokaryotic organisms in various phyla.

**SuppFigure 5.** Protein similarity map of clusters with experimentally-known TRs.

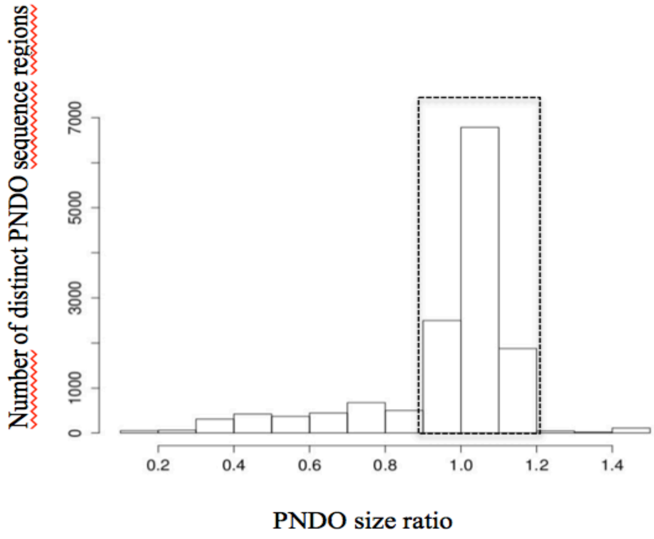


**SuppFigure 1. Distribution of PNDO size ratios from hmmer analysis with representative prokaryotic organisms.** For each hmmer alignment, the ratio between the observed PNDO alignment regions over the size of the PNDO profile was denoted as PNDO size ratio. Protein alignments with a PNDO size ration between 0.9 to 1.2 (which correspond to a PNDO size between 263 to 350 aa) represent ~78% of the total. Selected PNDO size ratios for subsequent analysis were boxed.

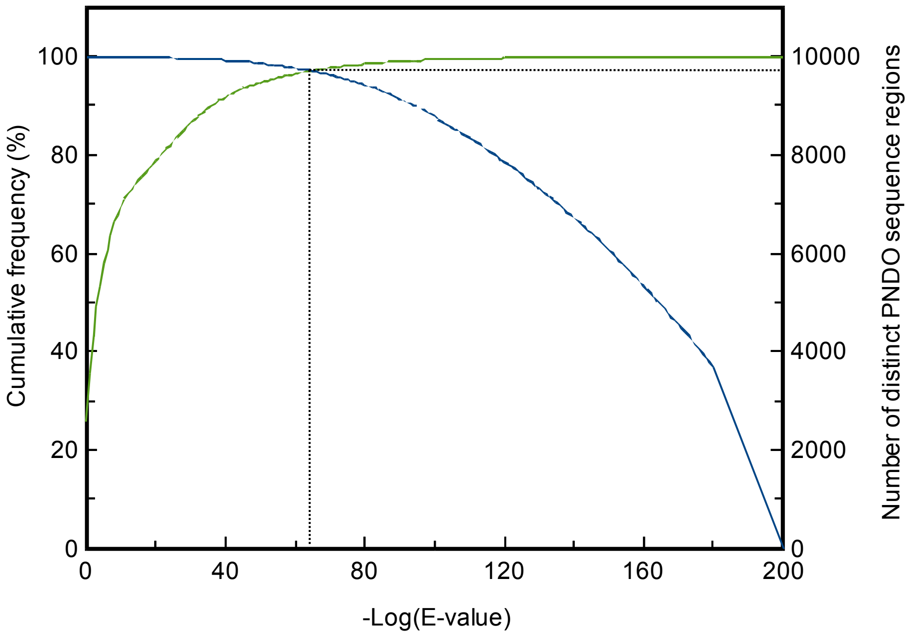


**SuppFigure 2. Distribution of pairwise alignments E-values between PNDO sequence regions from representative prokaryotic organisms.** The curve in green corresponds to the cumulative frequency of E-values from PNDO domains pairwise alignments (left Y axis). The blue curve indicates the number of sequences obtained after filtering with a given E-value (right Y axis). E-value of 0 was turn to $10^{-200}$ . The cross-point of the two curves of E-value ($10^{-64}$) defined the optimal E-value.

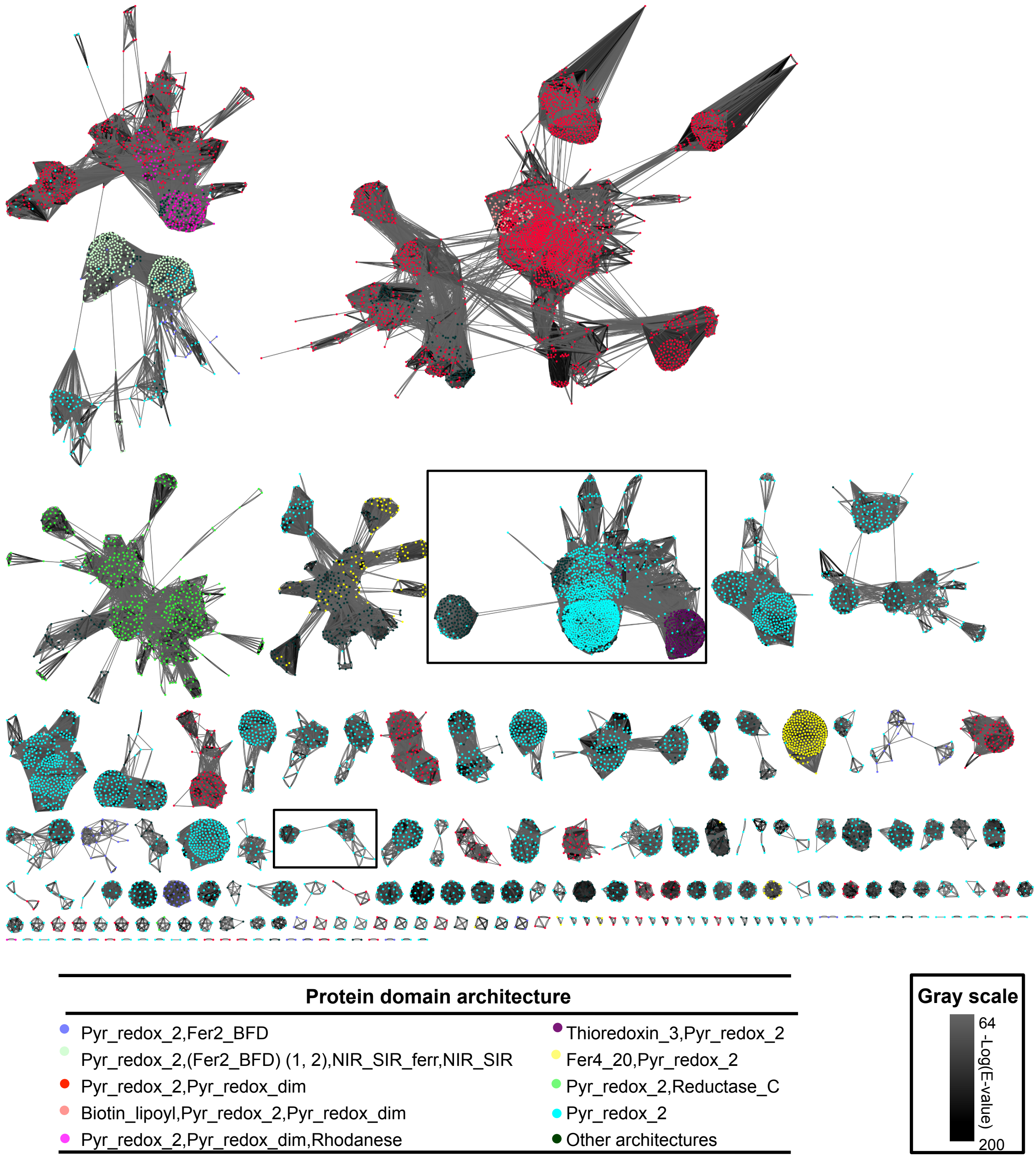


**SuppFigure 3. Protein similarity network (PSN) reconstructed with PNDO domains from representative prokaryotic organisms in various of architectures.** Sequences were represented by nodes, and the nodes shared the same protein domain architecture were in the same color. The edges were colored with a gray scale, and the darker the color was, the more significant similarity was. Clusters with experimentally-known thioredoxin reductases were boxed. DvTRi was located within the small box while other known TRs were located in the large box. The functional domain abbreviations (from Pfam database) are: Pyr_redox_2 (pyridine nucleotide-disulphide oxidoreductase, PNDO), Fer2_BFD (BFD-like [2Fe-2S] binding domain), NIR_SIR_ferr (nitrite/sulfite reductase ferredoxin-like half domain), NIR_SIR (nitrite and sulphite reductase 4Fe-4S domain), Biotin_lipoyl (biotin-requiring enzyme), Pyr_redox_dim (pyridine nucleotide-disulphide oxidoreductase, dimerisation domain), Rhodanese (rhodanese-like domain), Thioredoxin_3 (thioredoxin domain), Fer4_20 (dihydroprymidine dehydrogenase domain II, 4Fe-4S cluster), Reductase_C (reductase C-terminal).

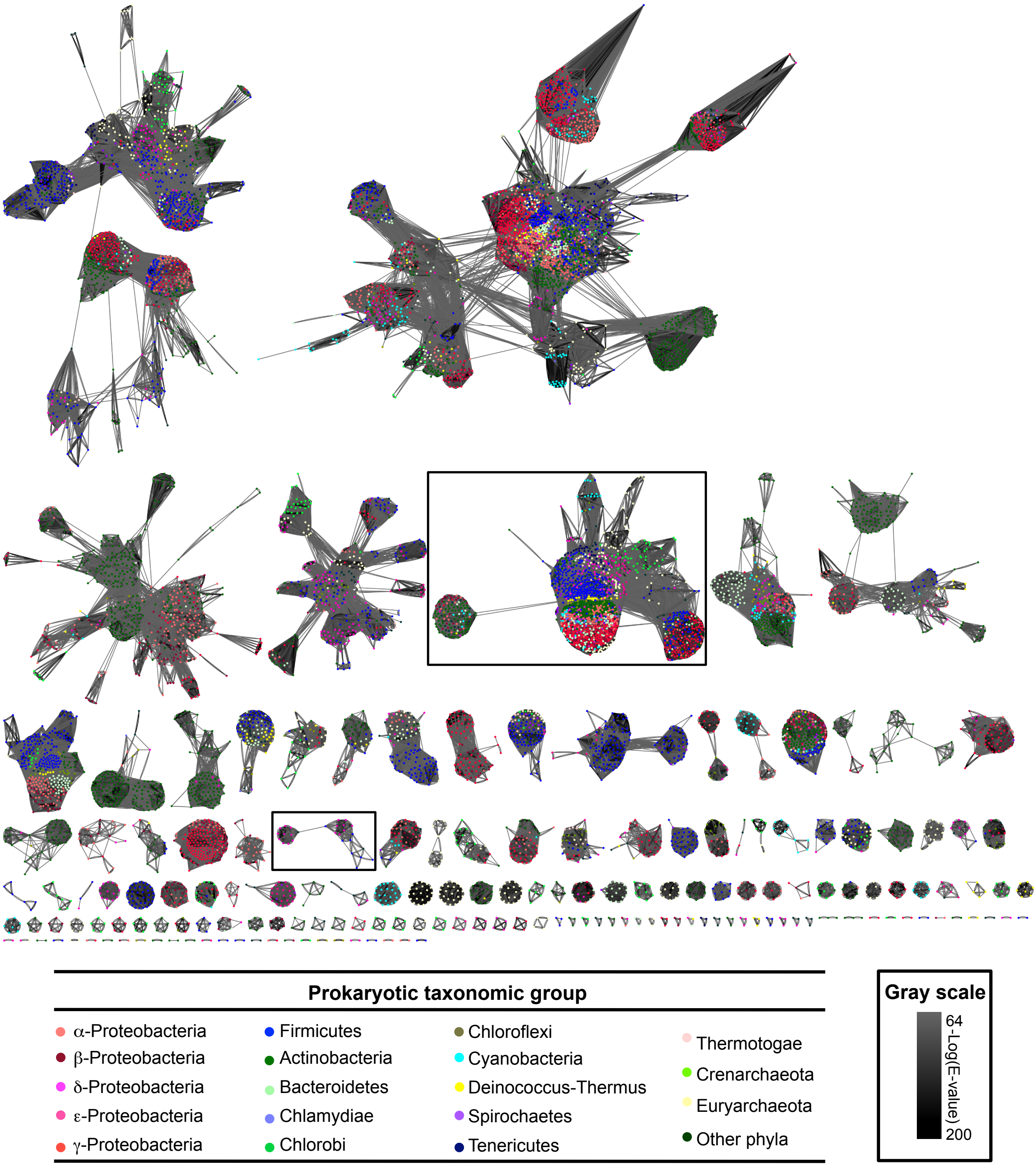


**SuppFigure 4. PSN reconstructed with PNDO sequence regions from representative prokaryotic organisms in various of phyla.** Sequences were represented by nodes, and the nodes shared the same prokaryotic taxonomic groups are in the same color. The edges were colored with a gray scale, and the darker the color was, the more significant similarity was. Clusters with experimentally-known thioredoxin reductases were boxed. DvTRi was located within the small box while other known TRs were located in the large box.

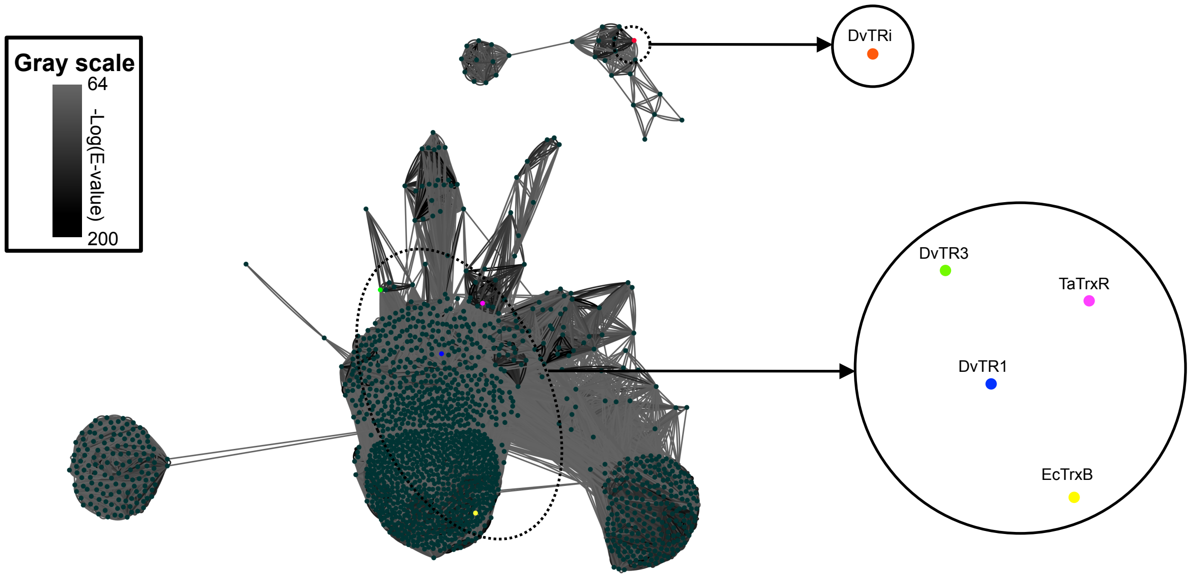


**SuppFigure 5. Protein similarity map of clusters with seed reference TRs**. Sequences were represented by nodes. TRs members were displayed by different color nodes. DvTRi: red; DvTR1: blue; DvTR3: green; TaTrxR: pink; EcTrxB: yellow. The edges were colored with a gray scale, and the darker the color was, the more significant similarity was. Network relationships between reference seed TRs were shown in circles.

**List of SuppTables**

**SuppTable 1.** List of experimentally-known thioredoxin reductase (TR) proteins used as references.

**SuppTable 2.** List of PNDO proteins with their domain features. (See independent Excell file).

**SuppTable 1.** List of experimentally-known thioredoxin reductase (TR) proteins used as references.

| TR reference | Gi Accession number | Protein length (aa) | Organism | Phylum | References |
|---|---|---|---|---|---|
| DvTRi | gi\|46579868\|ref\|YP_010676.1\| | 309 | *Desulfovibrio vulgaris* Hildenborough | Deltaproteobacteria | (Pieulle et al., 2011) |
| DvTR1 | gi\|46580247\|ref\|YP_011055.1\| | 307 | *Desulfovibrio vulgaris* Hildenborough | Deltaproteobacteria | (Pieulle et al., 2011) |
| DvTR3 | gi\|46578793\|ref\|YP_009601.1\| | 305 | *Desulfovibrio vulgaris* Hildenborough | Deltaproteobacteria | (Pieulle et al., 2011) |
| EcTrxB | gi\|16128855\|ref\|NP_415408.1\| | 321 | *Escherichia coli* K-12 | Gammaproteobacteria | (Waksman et al., 1994) |
| TaTrxR | gi\|16082022\|ref\|NP_394444.1\| | 319 | *Thermoplasma acidophilum* DSM 1728 | Euryarchaeota | (Hernandez et al., 2008) |

## List of Supplementary Figures

**Figure S1. A.** Variants of DvTRi and DvTrx1 analyzed on SDS-PAGE after purification. **B.** UV-visible spectra of TRi-C131S (left) and TRi-C134S (right).

**Figure S2.** Flowchart for finding DvTRs homologs.

**Figure S3.** Reduction of DvTRs with NAD(P)H.

**Figure S4.** Growth of the wild-type and *tri* mutant in lactate/sulfate medium.

**Figure S5.** Sequence alignment of DvTRi with EcTrxB.

**Figure S6.** Sequence logos of "active site" (**A**) and "NADPH binding" (**B**) regions obtained from HMM iterative profile for each TR groups.

**Figure S7.** Distribution of TR within prokaryotic taxonomic groups.

**Figure S8.** Structural comparison of NTR and non-NADPH dependent TRs.

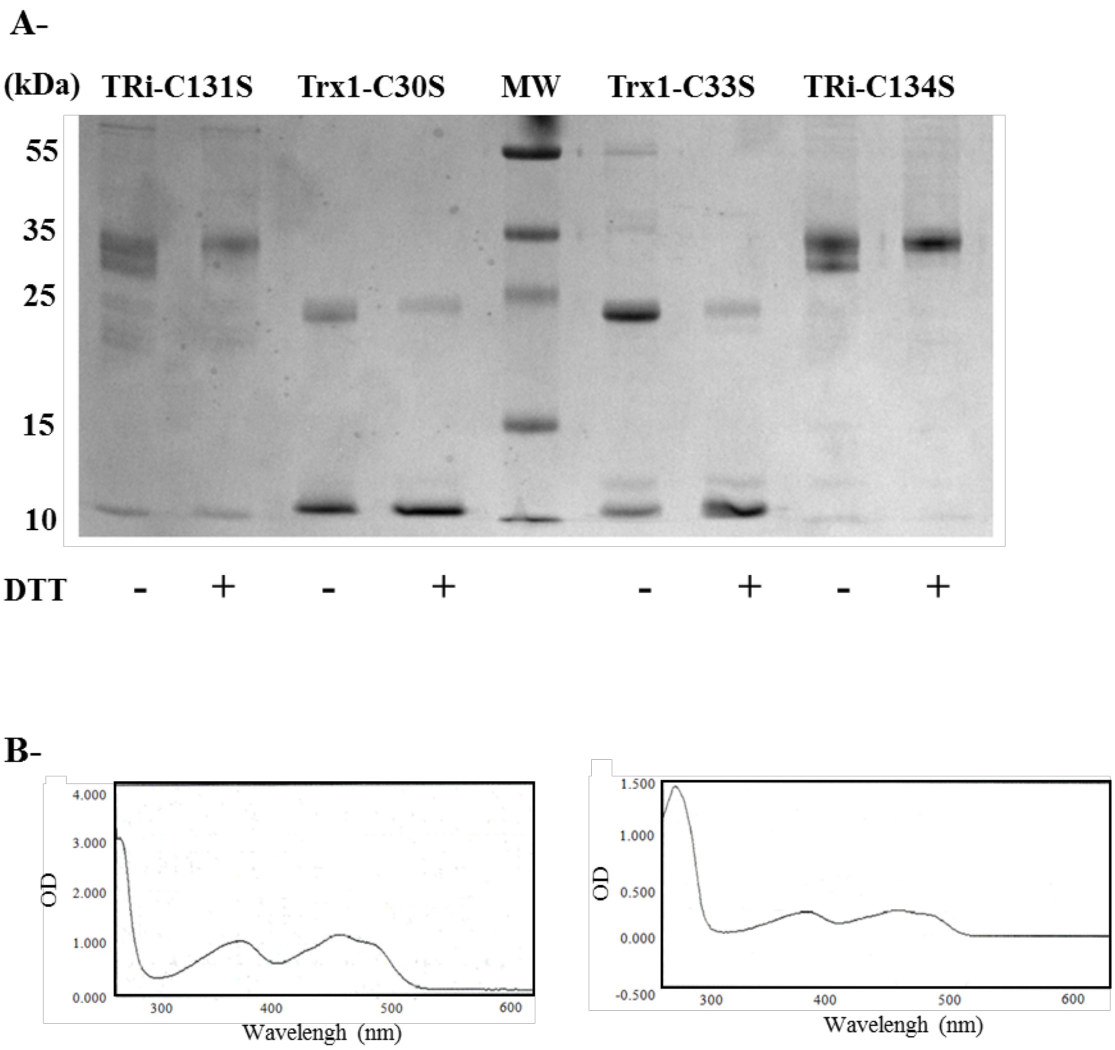


**Figure S1. A- Variants of DvTRi and DvTrx1 analyzed on SDS-PAGE after purification.** Protein samples were resuspended in a loading buffer containing or not DTT and separated on a SDS PAGE. MW: molecular weight standards. **B- UV-visible spectra of TRi-C131S (left) and TRi-C134S (right)**.

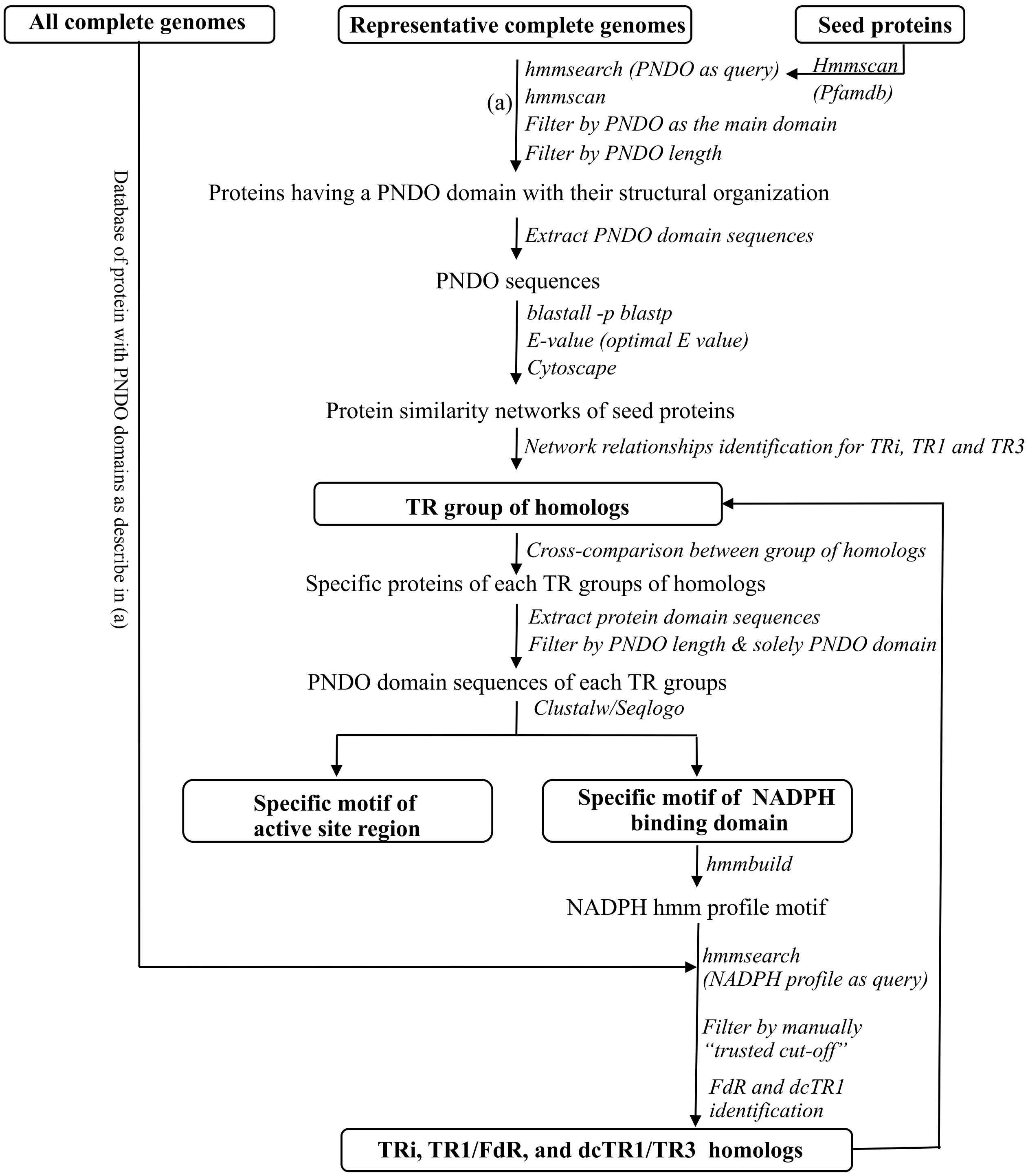


**Figure S2. Flowchart for finding DvTRs homologs.** PNDO, pyridine nucleotide-disulfide oxidoreductase.

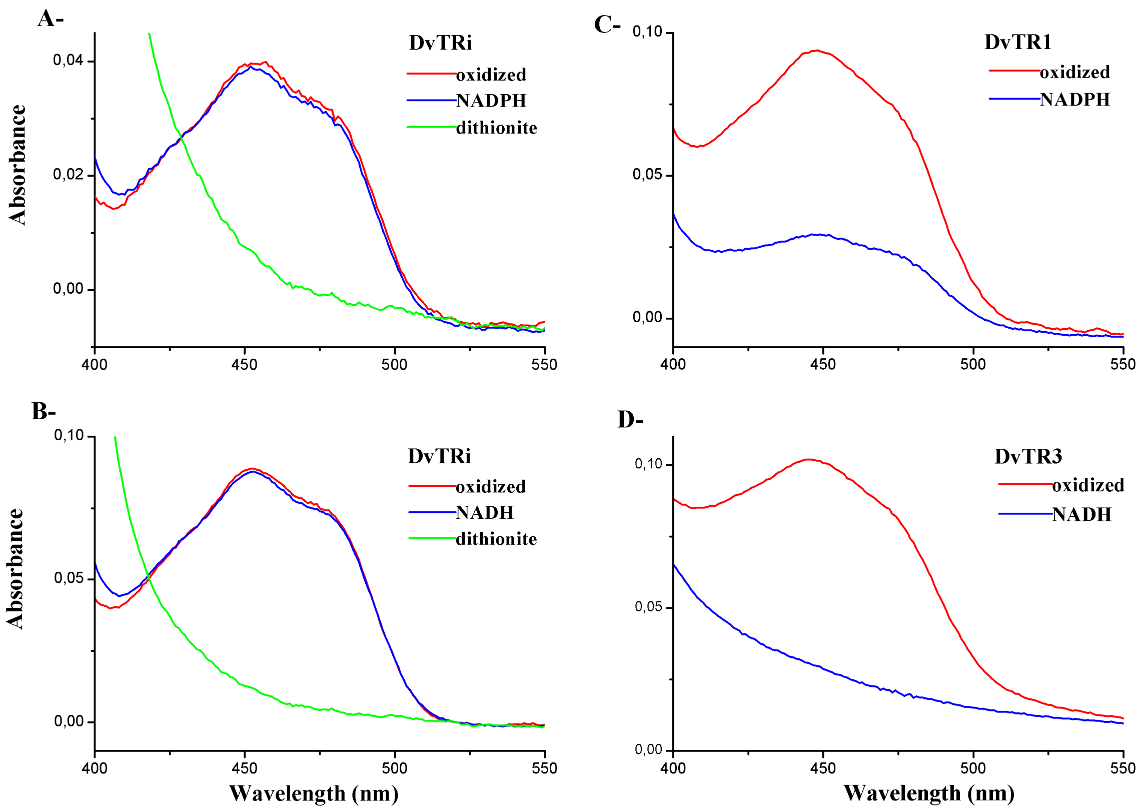


**Figure S3. Reduction of DvTRs with NAD(P)H. A-** and **B-** Visible spectra of DvTRi (8.6 µM (A) and 15.7 µM (B)) incubated with NAD(P)H (100 µM) at 25°C. Red line is without NAD(P)H, blue line is with NAD(P)H and green line is with an excess of dithionite. **C-** and **D-** Visible spectra of DvTR1 (17.6 µM) and DvTR3 (17.8 µM) incubated with NADPH (100 µM) and NADH (100 µM), respectively. Red line is without the reducer and blue line is with the reducer.

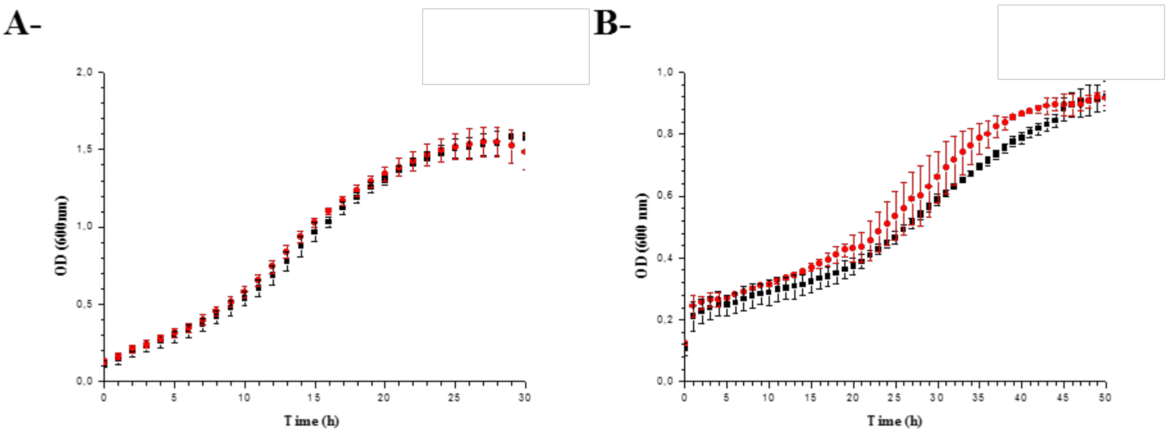


**Figure S4. Growth of the wild-type and *tri* mutant in lactate/sulfate medium. A-** Cell growth of the wild type (WT, black square) and *tri* mutant (red dot) in modified LS4DYE medium. **B-** WT (black square) and *tri* mutant (red square) in modified LS4D medium. The error bars indicate the standard deviations of the means from four independent growth curves. The doubling time of WT and *tri* mutant in rich media was 5.6 h ± 0.3 and 5.6 h ± 0.2, respectively, and in minimal medium 13.3 h ± 1.1 and 15.4 h ± 3.0. Doubling times were estimated by fitting the scatter plots on a logarithmic scale of optical density.

```
DvTRi           ---METRPLVIIGAGPAGLSAAVYTARAGIPTLVFGSAP---KVAGDYDIDNYFGFDETI 54
EcTrxB          MGTTKHSKLLILGSGPAGYTAAVYAARANLQPVLITGMEKGGQLTTTTEVENWPGDPNDL 60

DvTRi           TGRELIERGRRQAERFGAVLRDDRILGLHHGDDGGFRITTEAGETAACAIILATGVSRVR 114
EcTrxB          TGPLLMERMHEHATKF-ETEIIFDHINKVDLQNRPFRLNGDNGEYTCDALIIATGASARY 119

DvTRi           PGISNIADYEGKGVSYCVSCDGFFYRGLRVKVLGEGVFAANQALELLH-YTPHVSICTQG 173
EcTrxB          LGLPSEEAFKGRGVSACATCDGFFYRNQKVAVIGGGNTAVEEALYLSN-IASEVHLIHRR 178

DvTRi           KAASITPEFMTRLDEAG-----IAVDDRKIASLEGTPA----LSVLRYEDG---STEEAQ 221
EcTrxB          DGFRAEKILIKRLMDKVENGNIILHTNRTLEEVTGDQMGVTGVRLRDTQNSDNIESLDVA 238

DvTRi           GLFIAMG-EASSLDFAYTLGVERNGVFLGAD---SDQRTNIPGVFAAGDCTGG-FLQIAV 276
EcTrxB          GLFVAIGHSPNTAIFEGQLELENGYIKVQSGIHGNATQTSIPGVFAAGDVMDHIYRQAIT 298

DvTRi           AVGEGAKAARAAISYIKEECPFATSRRNTTTES 309
EcTrxB          SAGTGCMAALDAERYLDGLADAK---------- 321
```

**Figure S5. Sequence alignment of DvTRi and EcTrxB.** The FAD-domain of TRi is highlighted in blue, the pseudo NADPH-domain in red and the interdomain hinge region in green. The residues of the conserved motifs involved in FAD-binding are shown in bold and orange. Dv: *Desulfovibrio vulgaris* Hildenborough. Ec: *Escherichia coli*. Alignment was generated using the ClustalW program (version 2.0) from the EBI server *(*www.ebi.ac.uk/clustalw/).

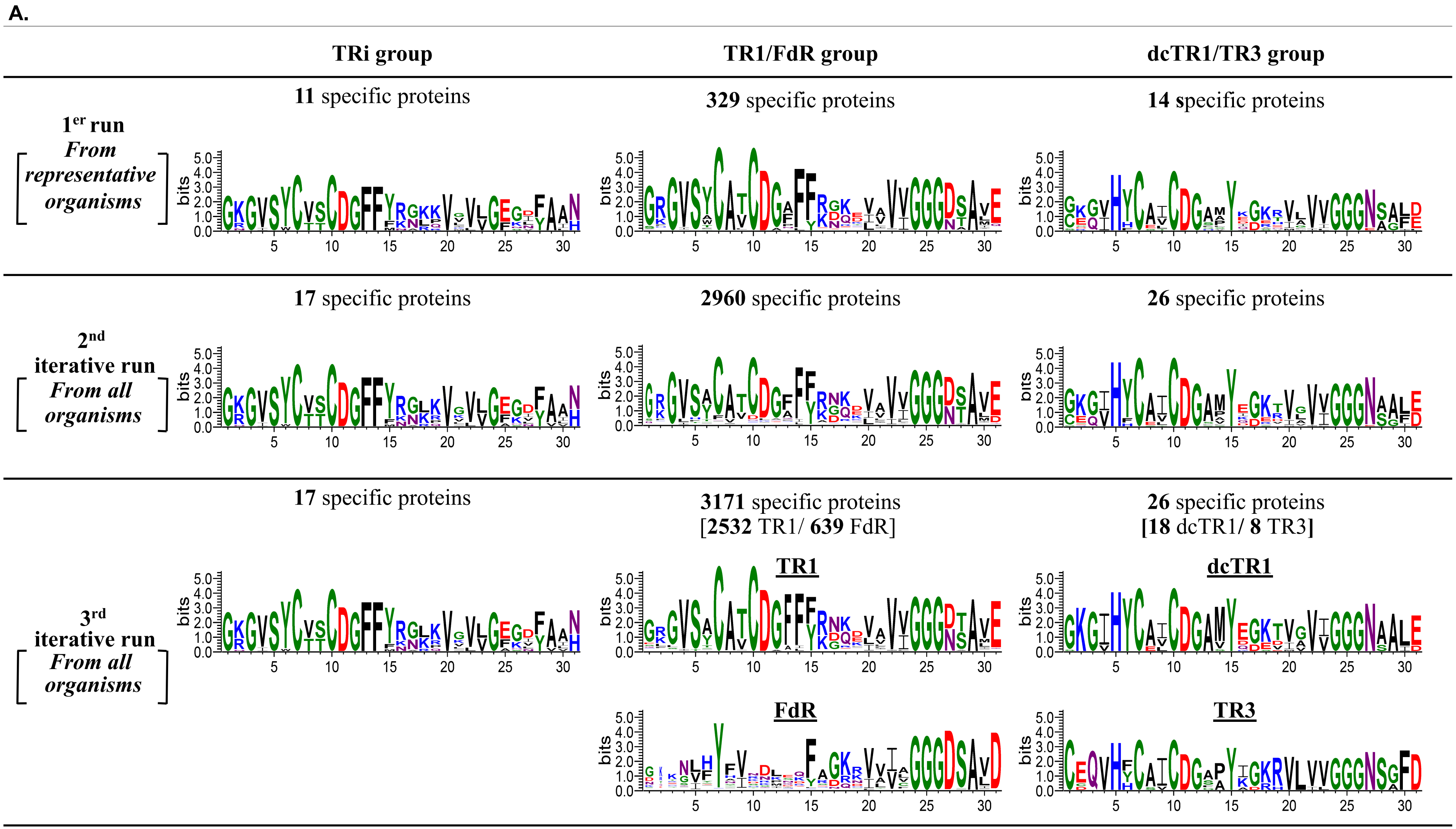
A.
TRi group
TR1/FdR group
dcTR1/TR3 group
1er run
From representative organisms
11 specific proteins
329 specific proteins
14 specific proteins
2nd iterative run
From all organisms
17 specific proteins
2960 specific proteins
26 specific proteins
3rd iterative run
From all organisms
17 specific proteins
3171 specific proteins
[2532 TR1/ 639 FdR]
26 specific proteins
[18 dcTR1/ 8 TR3]
TR1
FdR
dcTR1
TR3
bits
5
10
15
20
25
30

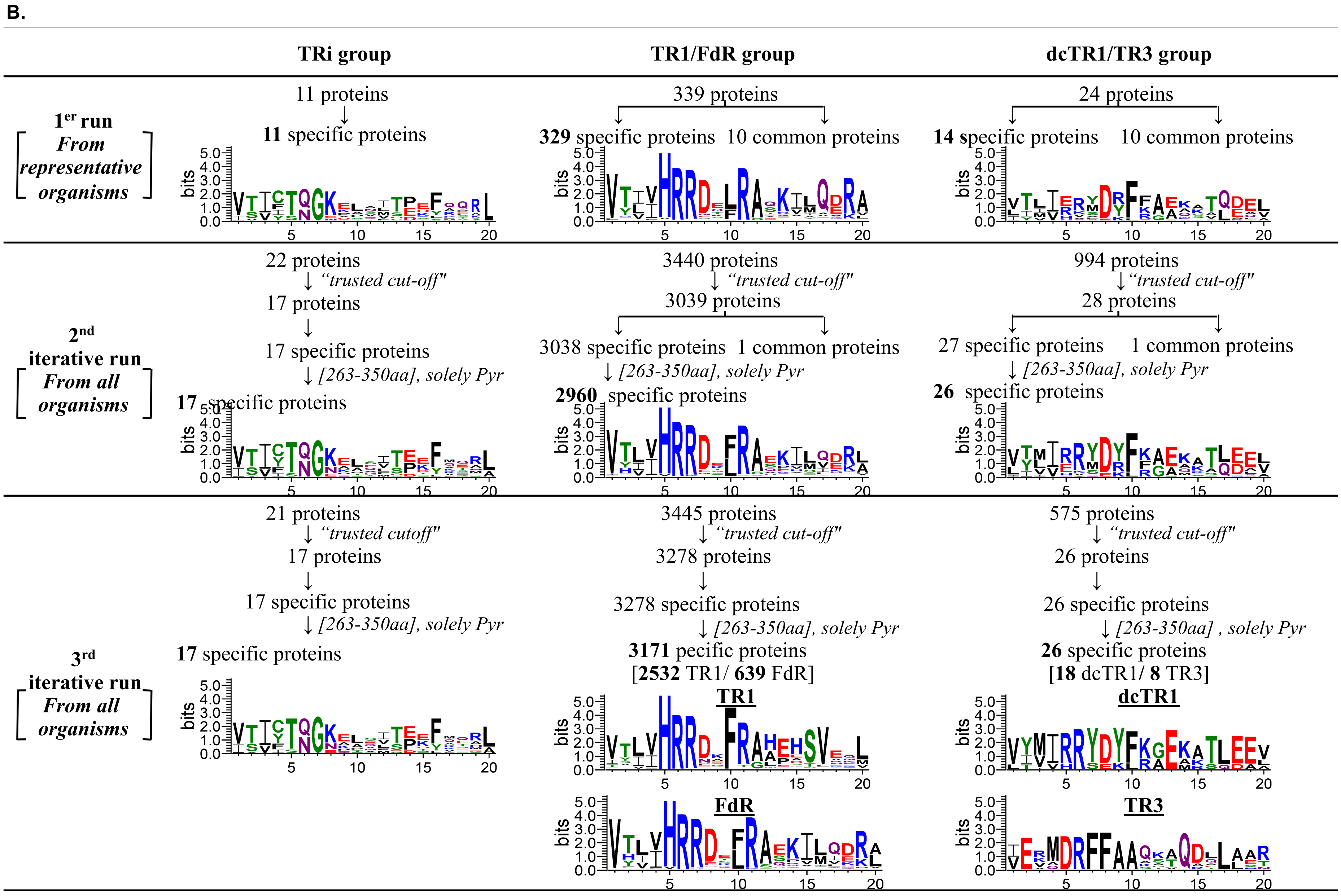


**Figure S6.** Sequence logos of "BoxA" (**A**) and "BoxB" (**B**) regions obtained from HMM iterative profile searches (2 iterative runs) for each TR groups. Details of the results are only displayed for **B**.

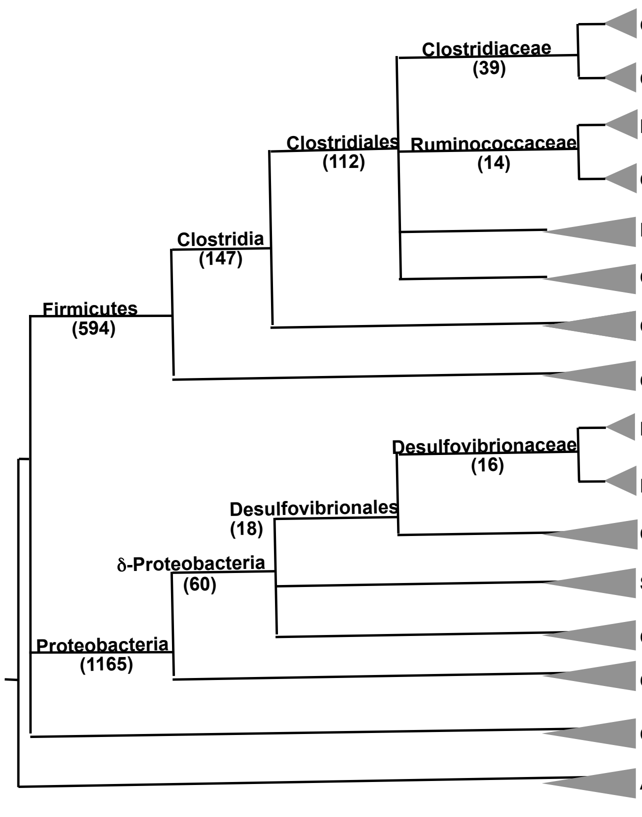


| | TRi | TR1 | dcTR1 | TR3 |
|---|---|---|---|---|
| Clostridium (34) | 2[2] | 20[18] | 17[17] | 0[0] |
| Other Clostridiaceae (5) | 0[0] | 5[5] | 0[0] | 0[0] |
| Ruminiclostridium (7) | 5[5] | 0[0] | 0[0] | 0[0] |
| Other Ruminococcaceae (7) | 0[0] | 6[5] | 0[0] | 0[0] |
| Lachnospiraceae (13) | 0[0] | 11[10] | 1[1] | 0[0] |
| Other Clostridiales (46) | 0[0] | 45[37] | 0[0] | 0[0] |
| Other Clostridia (35) | 0[0] | 25[22] | 0[0] | 0[0] |
| Other Firmicutes(447) | 0[0] | 457[432] | 0[0] | 0[0] |
| Desulfovibrio (14) | 8[8] | 16[14] | 0[0] | 7[5] |
| Lawsonia (2) | 0[0] | 2[2] | 0[0] | 0[0] |
| Other Desulfovibrionaceae (2) | 2[2] | 2[2] | 0[0] | 0[0] |
| Syntrophobacterales (4) | 0[0] | 5[4] | 0[0] | 1[1] |
| Other δ-Proteobacteria (38) | 0[0] | 37[36] | 0[0] | 0[0] |
| Other Proteobacteria (1105) | 0[0] | 1106[1063] | 0[0] | 0[0] |
| Other bacteria (852) | 0[0] | 676[643] | 0[0] | 0[0] |
| Archaea (165) | 0[0] | 110[110] | 0[0] | 0[0] |
| **OVERALL** | **17[17]** | **2532[2403]** | **18[18]** | **8[6]** |

**Figure S7. Distribution of TR within prokaryotic taxonomic groups.** Taxonomic subdivisions were taken from the NCBI taxonomic browser. The numbers within parentheses indicate the number of complete genome organisms available for this study. The numbers in table represent the number of TR proteins with number of organisms in brackets. Note that some organisms harbor paralogs or may contain TRs from distinct TR groups.

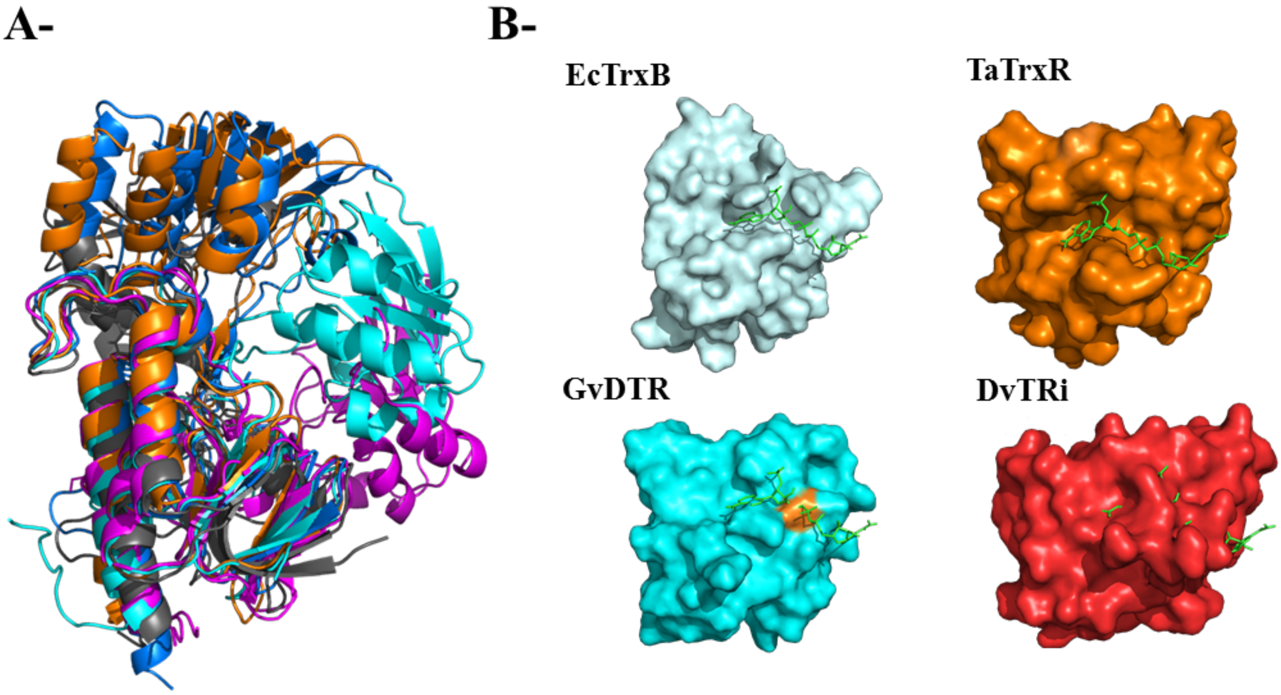


**Figure S8. Structural comparison of dependent and non-dependent NADPH TRs. A-** Superposition of EcTrxB FO (grey, PDB code: 1TDF), EcTrxB FR (blue, PDB code: 1F6M), TaTrxR (orange, PDB code: 3CTY) and GvDTR (cyan, PDB code: 5J60) forms with DvTRi (magenta). **B-** Comparison of solvent accessibility of pseudo- and NADPH-binding sites in TRs. All structures are represented in surface. NADPH analogues are depicted in green sticks. Asparagine 153 present in GvDTR (Buey et al., 2017) is depicted in orange. Ec: *Escherichia coli*; Ta: *Thermoplasma acidophilum*; Gv: *Gloeobacter violaceus*; Dv: *Desulfovibrio vulgaris* Hildenborough.

**List of Supplementary Tables**

**Table S1.** Primers DNA sequences, plasmids and strains used in this study.

**Table S2.** Crystallographic statistics

**Table S3.** List of TR1, FdR, dcTR1, TR3, and TRi protein homologs within prokaryotic genomes. (See independent Excell file).

## Table S1. Primer DNA sequences, plasmids and strains used in this study

| Primers | 5’ to 3’ Sequence |
|---|---|
| *For production of TR and Trx variants* | |
| TriC131Sdir | GGCAAGGGCGTATCCTACT**CG**GTGAGTTGCGACGGGTTC |
| TriC131Srev | GAACCCGTCGCAACTCACCGAGTAGGATACGCCCTTGCC |
| TriC134Sdir | GTATCCTACTGCGTGAGTT**CG**GACGGGTTCTTCTACC |
| TriC134Srev | GGTAGAAGAACCCGTCCGAACTCACGCAGTAGGATAC |
| Trx1C30Sdir | GACTTCTGGGCTCCCTGGT**CG**GGGCCGTGCCGTGCCATGG |
| Trx1C30Srev | CCATGGCACGGCACGGCCCCGACCAGGGAGCCCAGAAGTC |
| *For construction of deletion mutant and complementation studies* | |
| ATRi-fw | ATGGCGCGCCGTGGTCGAGCTGTTCCTCTC |
| ATRi-rev | GCACTAGTGCCACACACTTCAGACATGG |
| BTRi-fw | CGCCAATTGCTCAATCTGCACGCAAGGCAA |
| BTRi-rev | TCAGATCTTTCAGATACCGTCATAGACCCA |
| 1458dir2 | GACCTCACGGGACAGCGCATC |
| 1456rev2 | GATGTCGCGGGTGAGAAGTGTG |
| TRiG6PstIfw | AATCTGCAGGTGGTCGAGCTGTTCCTCTC |
| TRiG6KpnIrev | TTAGGTACCTCAAGACTCTGTCGTCGTGTTC |
| *For transcriptional analyses* | |
| Tr1-fw | GTGCTCATGACCTCCGAAAT |
| Tr1-rev | AATCCAGTCATCCCCTACCC |
| Tri-fw | TCTGTCGTCGTGTTCCTACG |
| Tri-rev | CACCAACATCCCTGGAGTCT |
| QrrsAL | TGGGGAGCAAACAGGATTAG |
| QrrsAR | CACATACTCCACCGCTTGTG |
| **Plasmids** | |
| *For construction of deletion mutant and complementation studies* | |
| pNOTCmΔ | (Vita et al., 2015) |
| pNOTCmΔ*tri* | This study |
| pBMG6 | (Rousset et al., 1998) |
| pG6*tri* | This study |
| *For production of TR and Trx variants* | |
| p119TRi | (Pieulle et al., 2011) |
| p119TRiC131S | This study |
| p119TRiC134S | This study |
| p119trx1 | (Pieulle et al., 2011) |
| p119trx1C30S | This study |
| p119trx1C33S | (Pieulle et al., 2011) |
| **Strains** | |
| *E. coli* DH5α | $F^-$ Φ80d*lac*ZM15 Δ(*lac*ZYA-*arg*F) U169 *rec*A *end*A1 *hsd*R17($r_{k-}$,$m_{k+}$) *pho*A *sup*E44 λ *thi*-1 *gyr*A96 *rel*A1 (Invitrogen) |
| *E. coli* TG1 | *sup*E *hsd*Δ5 *thi* Δ(*lac-pro*AB) $F^-$*tra*D36 *pro*$AB^+$ *lac*Iq *lac*ZÄM15 (Lab collection) |
| *D.vulgaris* Hildenborough | Wild-type (Lab collection) |
| *D.vulgaris*/pBMG6 | Wild-type/ pBMG6 $Tm^R$ $Gm^R$ (This study) |
| *D.vulgaris* Δ*tri* | Δ(*tri*) $Tm^R$ (This study) |
| *D.vulgaris* Δ*tri*/pG6*tri* | Δ(*tri*) / pG6*tri* $Tm^R$ $Gm^R$ (This study) |
| *D.vulgaris* Δ*tri*/pBMG6 | Δ(*tri*) / pBMG6 $Tm^R$ $Gm^R$ (This study) |

## Table S2. Crystallographic statistics

| **Data collection** | **DvTRi-Crystal1** |
|---|---|
| **PDB code** | **5NII** |
| beamline | ID29<br>(ESRF) |
| wavelength (Å) | 0.97625 |
| space group | *I*222 |
| unit cell parameters | a = 66.387 Å<br>b = 148.161 Å<br>c = 152.886 Å |
| resolution range (Å) | 47.4802-2.0 |
| $R_{sym}$ (%) | 4.9 [66.8] |
| I/σ | 16.24 [2.49] |
| CC (1/2) | 99.9 [82.1] |
| completeness (%) | 99.2 [99.7] |
| redundancy | 3.67 [3.77] |
| $N_{measured}$ | 186865 [25961] |
| $N_{unique}$ | 92563 [13784] |
| Refinement | |
| $R$ factor/ $R_{free}$ factor (%) | 18.71%/21.18% |
| No. atoms | 4847 |
| No. water molecules | 280 |
| average B-factor ($Å^2$) | 47.62 |
| Rmsd bonds (Å) | 0.018 |
| Rmsd angles (°) | 1.668 |
| Ramachandran plot: | |
| residues in most favorable region (%) | 98.98 % |
| residues in disallowed region (%) | 1.02% |

# REFERENCES

Altschul, S.F., Madden, T.L., Schaffer, A.A., Zhang, J., Zhang, Z., Miller, W., et al. (1997). Gapped BLAST and PSI-BLAST: a new generation of protein database search programs. *Nucleic Acids Res* 25**,** 3389-3402. doi:

Buey, R.M., Galindo-Trigo, S., López-Maury, L., Velázquez-Campoy, A., Revuelta, J.L., Florencio, F.J., et al. (2017). A New Member of the Thioredoxin Reductase Family from Early Oxygenic Photosynthetic Organisms. *Mol Plant* 10**,** 212-215. doi: 10.1016/j.molp.2016.06.019.

Finn, R.D., Coggill, P., Eberhardt, R.Y., Eddy, S.R., Mistry, J., Mitchell, A.L., et al. (2016). The Pfam protein families database: towards a more sustainable future. *Nucleic Acids Res* 44**,** D279-285. doi: 10.1093/nar/gkv1344.

Hernandez, H.H., Jaquez, O.A., Hamill, M.J., Elliott, S.J., and Drennan, C.L. (2008). Thioredoxin reductase from *Thermoplasma acidophilum*: a new twist on redox regulation. *Biochemistry* 47**,** 9728-9737. doi: 10.1021/bi8006753.

Mccutcheon, J.P., and Moran, N.A. (2012). Extreme genome reduction in symbiotic bacteria. *Nat Rev Microbiol* 10**,** 13-26. doi: 10.1038/nrmicro2670.

Mistry, J., Finn, R.D., Eddy, S.R., Bateman, A., and Punta, M. (2013). Challenges in homology search: HMMER3 and convergent evolution of coiled-coil regions. *Nucleic Acids Res* 41**,** e121. doi: 10.1093/nar/gkt263.

Pieulle, L., Stocker, P., Vinay, M., Nouailler, M., Vita, N., Brasseur, G., et al. (2011). Study of the thiol/disulfide redox systems of the anaerobe *Desulfovibrio vulgaris* points out pyruvate:ferredoxin oxidoreductase as a new target for thioredoxin 1. *J Biol Chem* 286**,** 7812-7821. doi: 10.1074/jbc.M110.197988.

Rousset, M., Casalot, L., Rapp-Giles, B.J., Dermoun, Z., De Philip, P., Belaich, J.P., et al. (1998). New shuttle vectors for the introduction of cloned DNA in *Desulfovibrio*. *Plasmid* 39**,** 114-122. doi: 10.1006/plas.1997.1321.

Shannon, P., Markiel, A., Ozier, O., Baliga, N.S., Wang, J.T., Ramage, D., et al. (2003). Cytoscape: a software environment for integrated models of biomolecular interaction networks. *Genome Res* 13**,** 2498-2504. doi: 10.1101/gr.1239303.

Vita, N., Valette, O., Brasseur, G., Lignon, S., Denis, Y., Ansaldi, M., et al. (2015). The primary pathway for lactate oxidation in *Desulfovibrio vulgaris*. *Front Microbiol* 6**,** 606. doi: 10.3389/fmicb.2015.00606.

Waksman, G., Krishna, T.S., Williams, C.H., and Kuriyan, J. (1994). Crystal structure of *Escherichia coli* thioredoxin reductase refined at 2 A resolution. Implications for a large conformational change during catalysis. *J Mol Biol* 236**,** 800-816. doi:

Zhang, Y., Zagnitko, O., Rodionova, I., Osterman, A., and Godzik, A. (2011). The FGGY carbohydrate kinase family: insights into the evolution of functional specificities. *PLoS Comput Biol* 7**,** e1002318. doi: 10.1371/journal.pcbi.1002318.
